# Characterization of Transcriptional Responses to Genomovirus Infection of the White Mold Fungus, *Sclerotinia sclerotiorum*

**DOI:** 10.3390/v14091892

**Published:** 2022-08-27

**Authors:** Connor J. Pedersen, Shin-Yi Lee Marzano

**Affiliations:** 1 Department of Biology and Microbiology, South Dakota State University, Brookings, SD 57007, USA; 2 United States Department of Agriculture/Agricultural Research Service, Toledo, OH 43606, USA; 3 Department of Horticulture, and Plant Science, South Dakota State University, Brookings, SD 57007, USA

**Keywords:** hypovirulence, genomovirus, mycovirus, *Sclerotinia sclerotiorum*, RNA-Seq

## Abstract

Soybean leaf-associated gemygorvirus-1 (SlaGemV−1) is a CRESS-DNA virus classified in the family Genomoviridae, which causes hypovirulence and abolishes sclerotia formation in infected fungal pathogens under the family Sclerotiniaceae. To investigate the mechanisms involved in the induction of hypovirulence, RNA-Seq was compared between virus-free and SlaGemV−1-infected *Sclerotinia sclerotiorum* strain DK3. Overall, 4639 genes were differentially expressed, with 50.5% up regulated and 49.5% down regulated genes. GO enrichments suggest changes in integral membrane components and transmission electron microscopy images reveal virus-like particles localized near the inner cell membrane. Differential gene expression analysis focused on genes responsible for cell cycle and DNA replication and repair pathways, ubiquitin proteolysis, gene silencing, methylation, pathogenesis-related, sclerotial development, carbohydrate metabolism, and oxalic acid biosynthesis. Carbohydrate metabolism showed the most changes, with two glycoside hydrolase genes being the most down regulated by −2396.1- and −648.6-fold. Genes relating to pathogenesis showed consistent down regulation with the greatest being SsNep1, SsSSVP1, and Endo2 showing, −4555-, −14.7-, and −12.3-fold changes. The cell cycle and DNA replication/repair pathways were almost entirely up regulated including a putative cyclin and separase being up regulated 8.3- and 5.2-fold. The oxalate decarboxylase genes necessary for oxalic acid catabolism and oxalic acid precursor biosynthesis genes and its metabolism show down regulations of −17.2- and −12.1-fold changes. Sclerotial formation genes also appear differentially regulated including a melanin biosynthesis gene *Pks*1 and a sclerotia formation gene *Sl*2 with fold changes of 3.8 and −2.9.

## 1. Introduction

Plant pathogenic fungi, which cause great economic losses worldwide and devastate food security and fiber supply and are oftentimes compounded by abiotic stresses, are expected to worsen with climate change. *Sclerotinia sclerotiorum* is a wide-reaching, highly pathogenic fungus responsible for upwards of $560 million in annual losses in soybean alone in the US [1], and weather conditions are important for carpogenic germination of sclerotia, the overwintering structure of the fungus. Considering the yields that are still incompletely protected by conventional means, such as the use of chemical pesticides and resistant cultivars, the deployment of biocontrol agents presents a promising approach to achieve the yield protection. Various studies have demonstrated the potential of disarming pathogenic fungi through the infection of mycoviruses as a biocontrol method specifically termed as viro-control [2,3]. Historically, viro-control has been well exemplified by the natural spread of Cryphonectria hypovirus 1 in Europe that controls the chestnut blight disease, and could be further developed for other disease controls of annual crops [4].

While some mycoviruses cause asymptomatic and latent infections [5], more mycoviruses are being identified to cause hypovirulence (reduced virulence) in their fungal hosts [6]. Several RNA and DNA viruses have been successfully rescued or purified to establish definitive cause-and-effect relationships of virus infection [7,8,9]. Furthermore, differential gene expression (DGE) between the virus free and virus-transfected cultures provides a clear picture on the genetic effects of the virus infection including identification of a *S. sclerotiorum* mycovirus which shows an alteration of sRNA accumulation [10] and another *S. sclerotiorum*-infecting mycovirus which attenuates growth showing deregulation of DNA replication/repair, carbohydrate metabolisms, translation, and virulence factors [11].

The diversity of mycoviruses identified so far have genome types in single-stranded RNA, double-stranded RNA, negative-sense RNA, and Circular Rep-Encoding Single-Stranded DNA (CRESS-DNA) forms [4]. Because of the extracellular transmission capability and stability in the environment, CRESS-DNA mycoviruses are good candidates to be developed as biocontrol pesticides [2]. The *S. sclerotiorum*-infecting mycovirus *Sclerotinia sclerotiorum* Hypovirulence-Associated Virus-1 (SsHADV-1) for example, which was discovered from a fungal isolate from *Brassica napus* tissue, has been shown to induce hypovirulence in *S. sclerotiorum* [9]. Hypovirulent SsHADV-1-infected *S. sclerotiorum* has also been shown to colonize plants as a potentially beneficial endophyte to induce plant disease resistance [2,12]. Transcriptomic study of SsHADV-1-infected *S. sclerotiorum* reveals changes in DNA replication and repair, expression of RNAi systems, and expression of virulence factors [11]. These transcriptomic changes within DNA replications and repair and RNAi silencing appear similar to the changes seen in plants during geminivirus, family of plant-infecting CRESS-DNA viruses, infection [13].

Fungal CRESS-DNA viruses are speculated to maintain functions similar to plant geminiviruses due to genetic homology in the coding of 2 major proteins: a capsid (CP) and a replication-associated protein (REP) [9,14]. Relatively better studied than gemonoviruses, plant geminivirus REP proteins are known to be multi-functional in their roles during infection (reviewed in [13]). REP in geminiviruses is responsible for binding and the initiation of rolling circle amplification of the viral genome [13,15] and CP creates the enclosed viral particle responsible for enveloping the genomic material [16] and shuttling the virus into the nucleus [17] by the nuclear localization signaling domain [18]. Within the nucleus, the viral particle uncoats and the viral REP protein relies on the host replicative machinery present during S phase to replicate by rolling circle amplification [19]. Often, geminivirus REP or REP-associated proteins will bind controllers of the plant entrance into S phase, such as plant retinoblastoma-related proteins, to force the cell cycle forward into the replication phase for the virus’s own benefit [20].

A CRESS-DNA virus, soybean leaf-associated gemygorvirus−1 (SlaGemV−1), was originally discovered through the metagenomic/metatranscriptomic sequencing of healthy soybean leaf tissue [21]. SlaGemV−1 shows promise in disarming pathogenic fungi in the family Sclerotiniaceae, including *S. sclerotiorum*, *B. cinerea*, and *M. fructicola*, to become non-pathogenic [7]. SlaGemV−1’s typical CRESS-DNA viral genome in a small, circular, 2-kb DNA form is similar to many other CRESS-DNA viruses that only encodes two proteins: CP and REP. The phenotype of SlaGemV−1-infected *S. sclerotiorum* DK3 is characterized by hypovirulence, a loss of pigmentation, and a complete abolition of sclerotia formation.

Our previous study showed that SlaGemV−1 induces hypovirulence in *S. sclerotiorum* and other fungal pathogens under family Sclerotiniaceae, and the heterologous expression in *B*. *cinerea* of REP alone was sufficient to cause a growth reduction and induce hypovirulence [7]. Because the genetic mechanism of hypovirulence is unknown, in this study we aimed to identify the effects of SlaGemV−1 infection on various fungal developmental and stress-related pathways, and the mechanisms involved in abolished production of sclerotia.

## 2. Materials and Methods

### 2.1. Preparation of Sclerotinia sclerotiorum Cultures and RNA Extraction

Virus-free (VF) and virus-transfected (VT) cultures of *S. sclerotiorum* strain DK3 were produced as described previously [22]. Fungal cultures were grown on cellophane laid on potato dextrose agar (PDA) at room temperature (~20 °C). Ten days old cultures were used to collect enough tissues because the growth on cellophane were slow, and it took ten days to reach the edge of a 9 mm Petri dish. Total RNAs were extracted from VF and VT cultures using the RNeasy Plant Mini Kit (Qiagen, Valencia, CA, USA) from tissue flash-frozen and pulverized by a bead-beating method in liquid nitrogen. RNA samples were extracted and eluted in DEPC-treated ddH_2_O and immediately stored at −80 °C.

### 2.2. TEM Imaging

PDA discs (5 mm) of mycelia from VF and VT were fixed in Karnovsky’s fixative in phosphate buffered 2% glutaraldehyde and 2.5% paraformaldehyde. Standard microwave procedures were used for embedding [23]. The tissue was subjected to ultrathin sectioning, stained and visualized for transmission electron microscope at the Materials Research Laboratory in University of Illinois Urbana-Champaign and more images of the same samples were taken with a TEM at the Electron Microscopy Facility, College of Medicine and Life Sciences, University of Toledo (Toledo, OH, USA).

### 2.3. Analysis of S. sclerotiorum Transcriptome

Libraries for RNA-Seq were constructed using TruSeq stranded mRNA kit (Illumina, San Diego, CA, USA). The RNA-Seq libraries were sequenced as single-end 100-nt reads on an Illumina NovaSeq and raw reads were uploaded to NCBI SRA and are accessible through the accession PRJNA643804. RNA-Seq analysis was analyzed using multiple programs, including BBDuk [24], FastQC [25], HISAT2 [26], Samtools [27], Subread [28], and cufflinks [29]. Differential gene expression analysis was done through the DESeq2 [30] and apeglm [31] was used to transform the data to log2. Both packages were used through R [32] where significance through DESeq2 was determined by fold change (FC) > 2 and FDR-adjusted *p*-value ≤ 0.05 (Benjamin-Hochberg). *Sclerotinia sclerotiorum* genome and annotation was acquired through the Joint Genome Institute MycoCosm resource [33]. Pathway analysis was done using the Kyoto Encyclopedia of Genes and Genomes (KEGG) database [34,35,36] and visualized through RStudio. FungiDB was used as a resource for determining GO enrichment tables [37] by separating Deseq2 results into up and down regulated genes and identifying the corresponding enrichments.

### 2.4. RT-qPCR

To validate the RNA-Seq results, primers were designed for genes with differential expression below the *p* ≤ 0.05 threshold but with marginal log_2_ fold changes or show great variability among the replications. Four biological replications of *S. sclerotiorum* with and without SlaGemV−1 infection were grown on PDA overlaid with a clear cellophane membrane (Research Products International, Mount Prospect, IL, USA) for 10 days. 100 mg of hyphal tissue was scraped from the surface, quickly frozen in liquid nitrogen, and homogenized for RNA extraction by a Quick-RNA^TM^ Plant Miniprep Kit by Zymo (Zymo Research, Irvine, CA, USA). RNA was extracted by manufacturer’s recommendation, eluted in nuclease-free H_2_O, treated with TURBO^TM^ DNase (Invitrogen, Waltham, MA, USA), aliquoted, and stored frozen at −80 °C. RT-qPCR was performed using a CYBRFast^TM^ 1-Step RT-qPCR Lo-ROX Kit by Tonbo (Tonbo Biosciences, San Diego, CA, USA) with 100 ng of template RNA per reaction on a CFX Connect Real-Time PCR Detection System^TM^ by Bio-Rad (Bio-Rad, Hercules, CA, USA) under the following conditions: 45 °C for 10 min, 95 °C for 2 min, 40 cycles of 95 °C for 5 s and 60 °C for 20 s. *Sclerotinia sclerotiorum* actin (SS1G_08733) was used as a reference gene and an inter-plate calibrator was ran for each subsequent plate. Relative expression was determined using CFX Maestro software by Bio-Rad.

## 3. Results

### 3.1. Sclerotinia sclerotiorum RNASeq Analysis

Results of adapter trimming, genome alignment, and feature annotation are detailed in Table S1. Illumina RNA-Seq adapters of 4 reps of virus-free and 4 reps of SlaGemV−1-infected *S. sclerotiorum* strain DK3 were trimmed by BBduk resulting in a loss of counts < 0.01% across all samples. Virus-free *S. sclerotiorum* RNAseq alignment showed 68.59–72.91% alignment against the *S. sclerotinia* genome for trimmed files of reads 24,854,390–26,379,211. Subread featureCounts alignment shows 49.4–74.9% assignments to the *S. sclerotiorum* annotation file. SlaGemV−1-infected *S. sclerotiorum* reads showed 65.61–67.77% alignment against the *S. sclerotinia* genome for trimmed files of reads 22,069,659–24,673,536. Subread featureCounts alignment shows 67.8–73.0% assignments to the *S. sclerotiorum* annotation file.

**Figure 1:**
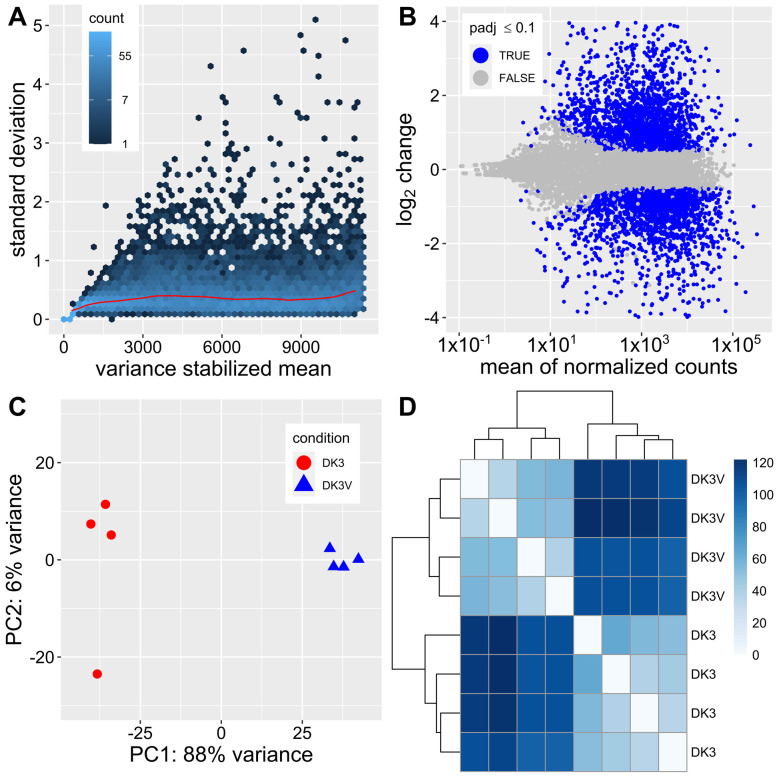
Differential expression analysis of DK3 vs. DK3-V where DK3 and DK3-V represent virus-free *S. sclerotiorum* strain DK3 and SlaGemV−1-infected DK3, respectively. (**A**) Variance stabilization of the normalized mean used for further DESeq2 analysis. (**B**) log_2_ fold change vs. mean of normalized counts found by variance stabilization. Initial visualization of padj ≤ 0.1 (Benjamin-Hochberg) where positive log change indicates genes which have been up regulated in the virus-infected samples shows an abundance of differentially expressed genes. (**C**) Principal component analysis of variance between DK3 and DK3-V with principal components of 88% and 6% showing the clustering of DK3 and DK3V samples together. (**D**). Distance matrix displayed as a heatmap of total gene expression between DK3 and DK3-V shows clear differences in expression between samples DK3 and DK3-V.

4639 genes were found to be differentially expressed between virus-free (DK3) and SlaGemV−1-infected (DK3-V) *S. sclerotiorum* cultures using the feature counts table input into DESeq2. 2294 (49.5%) genes were shown to be down regulated, and 2345 (50.5%) genes were shown to be up regulated by viral infection with a cutoff of padj ≤ 0.05 (Benjamin-Hochberg). Results of the DESeq2 differential expression analysis for *S. sclerotiorum* with or without SlaGemV−1 infection are visualized in Figure 1A displays the normalization of the reads by method of variance stabilization which would be used for further analysis. Normal transformation and regularized log transformation methods were also tested (Figure S1). Variance stabilization offers the most constant rate of standard deviation compared to the shifted log and regularized log transformations. Figure 1B shows differential expression cutoff based on padj ≤ 0.1 which was adjusted during downstream analysis to include only padj ≤ 0.05; positive log_2_ change correlates to an up regulation of genes caused by SlaGemV−1 infection while a negative log_2_ change correlates to a down regulation of genes caused by SlaGemV−1 infection. Figure 1C displays principal component analysis with component variances of 88% and 6% with DK3 and DK3-V samples clustered together, respectively. Figure 1D shows the sample distance clustering visualized by a heatmap. RNA-Seq analysis visualized in Figure 1A–D reveal significant differential expression in samples of *S. sclerotiorum* virus-free and *S. sclerotiorum* infected with SlaGemV−1 mycovirus. Sample distances show similarities and dissimilarities and clustering of the virus-infected and virus-free total RNA samples. As a confirmation, analysis of differential expression using a different program, EdgeR [38], yields similar results (Figure S2).

### 3.2. GO Ontology Enrichment and Viral Distribution

The GO enrichment between the up and down regulated genes shows major differences in potentially affected pathways. Five molecular functions were up regulated while 12 molecular functions were down regulated; similarly, GO biological functions also saw more down regulation with 33 GO terms up regulated and 65 GO terms down regulated. The trend continues with GO cellular component enrichments with 15 up regulated GO terms and 20 down regulated GO terms. Changes in GO enrichment are listed in Tables S2–S4 for GO molecular function, GO biological processes, and GO cellular components, respectively. Go terms of particular interest included GO molecular functions DNA-related ontology networks among the up regulated genes (GO:0140097, GO:0003677, GO:0008094, and GO:0003678) and polysaccharide/cellulose binding activities among the down regulated genes (GO:0030248, GO:0030247, GO:0030247, and GO:0030246). In GO biological processes, enrichments were found among the up regulated genes for cell cycle and DNA replication-related processes (GO:0007049, GO:0006259, GO:0006260) and enrichment among the down regulated genes for small molecule metabolic processes (GO:0044281) as well as an enrichment for oxoacid metabolism (GO:0043436) which indicates changes in the biosynthesis and metabolism of oxalic acid and oxaloacetate. In GO cellular component analysis, among up regulated genes enrichments for chromosome, condensed chromosome, chromosomal region, mismatch repair, and replication fork are all enriched (GO:0005694, GO:0000793, GO:0098687, GO:0032300, and GO:0005657), which represents changes in cell cycle, DNA replication/repair, and chromatin remodeling. Enrichments of genes relating to the host cell cycle and DNA replication and repair pathways indicate the entrance into viral replication pathways was affected. Cellulose-binding domains enriched amongst down regulated genes, common to pathogenicity-determinate genes of *S. sclerotiorum* [39,40], also indicates a larger loss of function among other pathogenicity determinants.

**Figure 2:**
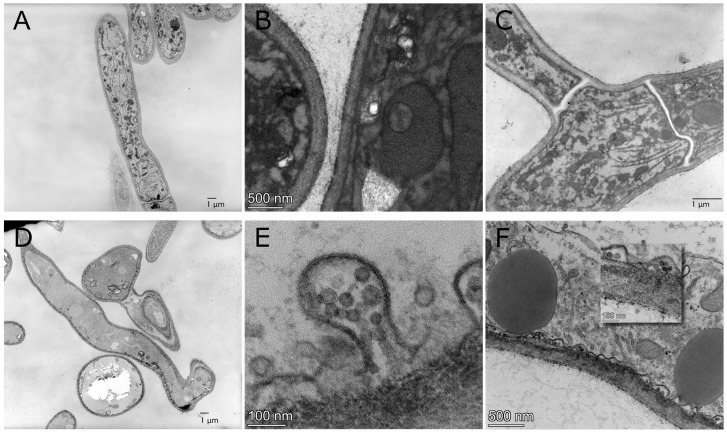
Transmission electron microscopy images of virus-free *S. sclerotiorum* DK3 (**A**–**C**) and SlaGemV−1-infected *S. sclerotiorum* DK3 (**D**–**F**). (**A**,**D**) show an apparent change in morphology of the fungal cell with SlaGemV−1 infection. In (**E**,**F**), 20–30 nm icosahedral particles can be seen and contained in vesicles along the inner cell membrane. These particles and vesicles are absent in virus-free samples in (**B**,**C**).

Of particular interest in cellular component analysis, the GO enrichment GO:0016021 (integral part of membrane) suggests that changes within the fungal cell membrane was apparent. Therefore, TEM imaging was utilized to confirm the differences between cell membranes of virus-free and SlaGemV−1-infected *S. sclerotiorum*. Figure 2 shows the images captured for virus-free (Figure 2A) and SlaGemV−1-infected (Figure 2D) samples of *S. sclerotiorum* DK3 under a lower magnification. At 3000×, a morphological change of the shape of the fungal cell can be seen. Focusing closer to the membrane reveals potential virus-like particles present in the virus-infected samples (Figure 2E,F), which are absent in virus-free samples (Figure 2B,C). SlaGemV−1 virus particles have been estimated to be 20–25 nm in diameter in a previous study [7] and Figure 2E,F show icosahedral-like particles which appear concentrated within vesicles adjacent to the inner cell membrane.

### 3.3. RT-qPCR Confirmation of Genes with High Sequence Bias

Primers were designed and checked for linearity for genes which showed high variability of differential expression levels between the four biological replications. Genes tested include: Dss1, DNA Pol ε subunit 2, Ago-4, β-fructofuranosidase, cellobiohydrolase, Mcm4, PCNA, Qde1, RNase H2 subunit C, Rrp3, Sad1, Top3, and ubiquitin-conjugating enzyme E2 H (Table S14). These genes with high variability among samples all showed no differential expression during RT-qPCR (Table S15).

### 3.4. Cell Cycle, DNA Repair/Replication, and Ubiquitin Proteolysis Modulation

**Figure 3:**
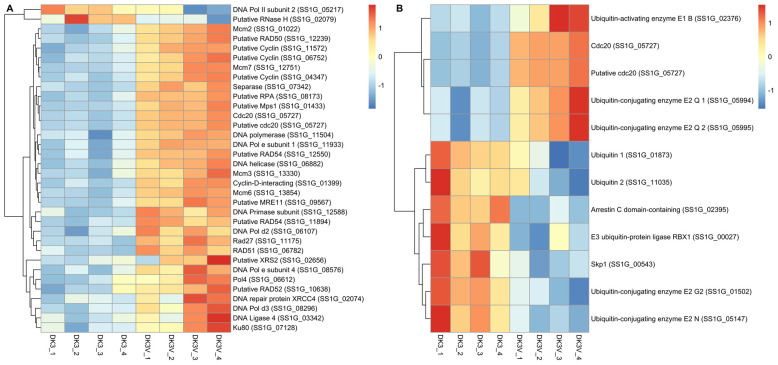
(**A**) Cell cycle and DNA replication/repair (**B**) and ubiquitin proteolysis genes were investigated for potential differential regulation in presence of SlaGemV−1 infection in *S. sclerotiorum*. Distance matrices of the differential expression analysis were visualized as heatmaps. Genes relating to cell cycle controls and DNA replication and repair are seen largely up regulated in presence of SlaGemV−1. Likewise, genes relating to the up regulation of ubiquitin proteolysis pathway are also up regulated during viral infection.

Recognizing that plant geminiviruses, which are similar in structure to fungal genomoviruses, often bind to/modulate proteins in the cell cycle, *S. sclerotiorum* genes annotated by the KEGG cell cycle pathway were investigated for differential expression. Figure 3A and Table S5 show differential expression of genes relating to DNA replication/repair and the cell cycle. Most genes relating to the proliferation of the cell cycle and the replication and repair of DNA show an up regulation in SlaGemV−1-infected culture. Genes relating to both homologous recombination and non-homologous end-joining show differential expression. Additionally, genes directly relating to DNA replication are seen differentially regulated. Slight down regulation of fold changes in an RNase H (−7.6-fold change) and DNA Pol II subunit 2 (−1.6-fold change) are observed, while several genes relating to the cell cycle, DNA replication, and DNA repair are significantly up regulated, including DNA Pol ε subunits 1 (4.3-fold change) and 4 (1.6-fold change). RAD27 (2.2-fold change), RAD50 (2.2-fold change), RAD51 (2.0-fold change), RAD52 (2.1-fold change), and RAD54 (2.3-fold change), genes important for homologous and non-homologous end-joining pathways, are seen with slight up regulation as well as DNA Polymerase 4 (1.8-fold change) and DNA ligase 4 (1.4-fold change). Putative cell cycle control proteins such as cyclins (8.3-, 3.7-, 2.8-fold changes), a cyclin-interacting protein (5.9-fold change), separase (5.2-fold change) and cell division cycle 20 protein (5.5-fold change) all see up regulation. Figure S3 displays a larger collection of cell cycle and DNA replication/repair genes before annotation and filtering, suggesting a broader change in differential expression within these pathways.

Further, Hanley-Bowdoin et al. describe that ubiquitin proteolysis pathways are modulated by plant geminiviruses, which are further investigated in the RNA-Seq [13]. Ubiquitin-related enzyme E2 (2.1-fold change) show some differential expression in presence of SlaGemV−1, while the ubiquitin enzyme E3 cdc20, which also acts as a cell cycle regulator, shows distinct down regulation of −5.5 fold. An arrestin C-domain containing protein also shows differential expression of −1.2 fold (Figure 3B, Table S6).

### 3.5. Silencing and Methylation-Related Pathways

**Figure 4:**
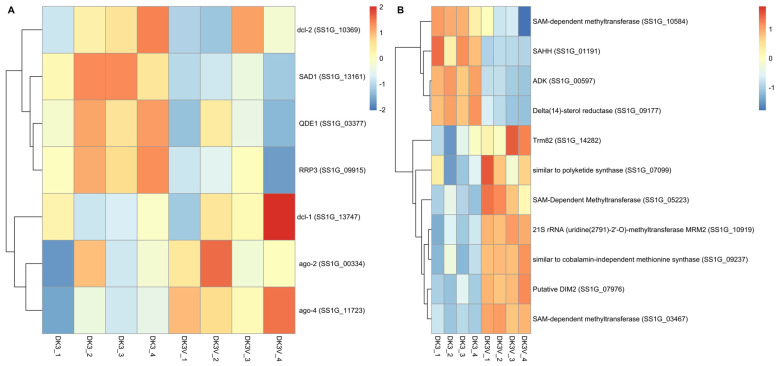
RNAi silencing (**A**) and methylation-related (**B**) genes were investigated for potential differential regulation in presence of SlaGemV−1 infection in *S. sclerotiorum*. Distance matrices of the differential expression analysis were visualized as heatmaps. Neither dicer nor argonaut-2 show differential expression, while argonaut-4 is upregulated and all 3 RdRps are downregulated. The *S*-Adenosyl-*L*-methionine biosynthesis protein ADK is down regulated in SlaGemV−1-infected samples as well as SAHH, which has been shown as a target of plant geminiviruses to prevent viral genome methylation. Other methylation genes, including SAM-dependent methyltransferases, show differential expression.

To investigate whether *S. Sclerotiorum* activates antiviral RNA silencing upon infection by SlaGemV−1 via transcriptional up-regulation of key RNA silencing genes as in *C*. *parasitica* [41], genes related to RNA silencing were further profiled as seen in Figure 4A and Table S7. No DGE was seen in either dicer gene or in argonaute-2 and 4. A slight differential expression of the three RdRp genes of *S. sclerotiorum* were detected by RNA-Seq, and a validation by RT-qPCR found no significant DGE, consistent with the RNA-Seq results (Table S15).

Similarly to effects seen in plant geminiviruses, defense-related methylation systems may also be differentially expressed [13]. Putative *S. sclerotiorum* adenosine kinase (ADK) necessary for S-adenosyl methionine (SAM) synthesis shows down regulation with a FC of −3.5. Putative *S. sclerotiorum* S-adenosyl homocysteine hydrolase (SAHH) required for transcriptional gene silencing (TGS) is also seen down regulated (−3.2-fold change). Moreover, a putative cysteine-specific methylase DIM2 is seen up regulated (3.5-fold change).

### 3.6. Effects on Pathogenesis and Metabolism-Related Genes

Xu et al. and Xia et al. describe genes necessary for the pathogenesis of *S. sclerotiorum* which were searched for in the RNA-Seq to determine a correlation between differential expression and pathogenesis [42,43]. These genes were referenced to build a list of pathogenesis-related and sclerotial formation-related genes. Additionally, carbohydrate metabolism and oxoacid-related pathways were investigated due to their GO ID enrichments found above.

**Figure 5:**
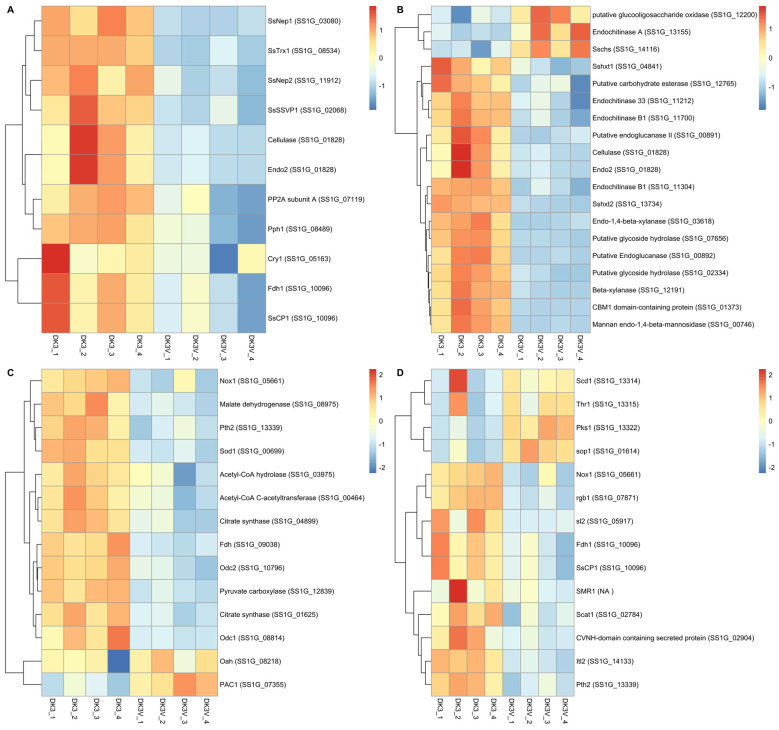
(**A**) Pathogenesis-related, (**B**) Carbohydrate biosynthesis and metabolism, (**C**) oxalic acid biosynthesis, metabolism, and citric acid synthesis, (**D**) and Sclerotial formation genes were investigated for potential differential regulation in presence of SlaGemV−1 infection in *S. sclerotiorum*. Distance matrices of the differential expression analysis were visualized as heatmaps. Pathogenesis-related genes identified were all down regulated, while most sclerotiral formation genes appear down regulated.

Pathogenesis-related genes were seen down-regulated including Endo2, SsNep1, SsNep2, SsCP1, SsSsvp1, SsTrx1, and Pph1 (−12.1, −4,555, −4.6, 4.7, −14.7, −3.6, −1.7-fold changes). A distance heatmap of expression changes is shown in Figure 5A and fold changes and *p-*values are shown in Supplemental Table S9. Furthermore, differential expression of polysaccharide metabolism, including cellulose and chitin metabolism, are visualized in Figure 5C. Genes relating to the metabolism of cellulose and chitin were also shown to have significant differential expression (Table S11). Chitin biosynthesis remains marginally up regulated (Sschs at 1.5-fold change) while down regulation (endochitinase 33: −4.5 and endochitinase B: −9.3-fold change) of chitinase genes are also observed, while endochitinase A remains up regulated at 3.2-fold. Two putative glycoside hydrolase genes were seen down regulated −2396.1 and −648.6-fold.

Oxalic acid biosynthesis and metabolism were both investigated for differential expression and described in Figure 5D and Table S12. The oxalic acid biosynthesis gene Oah does not show any differential expression. However, oxalic acid decarboxylase genes (Odc1 and 2) do both show down regulations of −17.2 and −12.1 fold, respectively. Further, genes Sod1 and Pac1 also show differential expressions of −2.3 and 4.2-fold. Genes relating to the biosynthesis of oxaloacetate as well as its conversion to citric acid show differential expression including malate dehydrogenase, pyruvate dehydrogenase, acetyl-CoA C-acetyltransferase, and two citrate synthases with fold changes of −1.9 and −2.7, −2.2, −2.8, and −2.7-fold change.

### 3.7. Effects on Sclerotial Formation and Melanization Genes

Sclerotial formation genes also appear differentially expressed during SlaGemV−1 infection, although two melanin biosynthesis genes (Scd1 and Thr1) do not appear differentially expressed, the melanin biosynthesis gene Pks1 shows a 14.4-fold change increase in expression. Genes which result in abolition of sclerotial formation when silenced including Pth2 and sl2 which appear down regulated (−2.0 and −7.4 fold) and sop1 which is up regulated 5.5-fold. Some genes which when deleted reduce, but not abolish, sclerotial formation are also down regulated: Itl2 and Smr1 (−5.4 and −2.7 fold) while Pac 1 was up regulated 4.2-fold.

## 4. Discussion

### 4.1. Viral Particles Appear to Localize along the Inner Cell Membrane

Gene ontology analysis (term GO:0016021 for integral part of membrane) being significantly enriched and up regulated due to SlaGemV−1 infection suggests a change in membrane morphology which is consistent with our TEM findings in Figure 2. A deformed cell membrane under SlaGemV−1 infection was noticeable. Further, plant geminiviruses exhibit the ability to bind to the cytoplasmic-facing membranes of their hosts to trigger horizontal transfer of viruses between cells [18]. The localization of virus-like particles near the cell membrane in Figure 2 suggests that SlaGemV−1 CP or REP may contain a membrane-binding motif responsible for intercellular transfer of virus particles.

### 4.2. Cell Cycle, DNA Replication/Repair, and Ubiquitylation

Gene ontologies relating to DNA-binding, catalytic activity acting on DNA, cell cycle, chromosomal organization and chromosome localization being enriched amongst the up regulated gene lists may indicate modifications of the cell cycle in presence of SlaGemV−1 infection. Effects on *S. sclerotiorum* DNA synthesis/cell cycle pathways were hypothesized to occur as similar changes in genetic expression are seen during plant infection by geminiviruses [13]. Similar ontology results can be seen in both GO biological process and cellular component enrichments. Furthermore, as potential disruptions to the cell cycle as described by Hanley-Bowdoin et al., under geminivirus infection, are observed in GO enrichments, ubiquitin proteolysis and methylation pathways would also be investigated for their connection to geminivirus infections [13].

Gene ontology analysis shows positive enrichment of cell cycle and DNA replication and repair during SlaGemV−1 infection (GO:0007049, GO:0006281, and GO:0006260) as well as cellular component enrichment of chromosomal and replications regions which may indicate symptoms of chromatin remodeling (GO:0005694, GO:0000793, GO:0098687, GO:0032300, and GO:0005657), and similar to geminivirus infection, alterations in the expression of DNA replication and repair can be observed during SlaGemV−1 infection [44]. DNA replication genes show differential expression including a down regulated RNase H gene and an up regulated DNA polymerase ε subunit 1; as well as homologous recombination genes RAD 51 and RAD 52. In geminivirus, Rep interacts with proliferating cell nuclear antigen (PCNA) to be loaded onto ssDNA and the replication protein A. PCNA is an important, conserved, protein which interacts within DNA replication, repair, and cell cycle pathways [13]. Here, we do not see changes in the expression of PCNA, but we do see expression changes in other Rep-binding candidates. Geminivirus rep may also bind to RAD54, which is involved in homologous recombination, which might have a role in viral replication mediated by recombination-dependent replication [13]. Two putative RAD54 proteins can be seen up regulated > 2-fold in presence of SlaGemV−1 infection. Further, the MRX complex, an important protein complex for DNA double-stranded break repair [45] during both homologous recombination and non-homologous end-joining, shows up regulation in two of its three known protein constituents RAD50 and MRE11, with XRS2 not annotated in *S. sclerotiorum*. Further genes relating to homologous recombination (RPA, DNA Pol, DNA Pol δ2, DNA Pol δ3) and non-homologous end-joining (KU80, RAD27) also see up regulation in presence of SlaGemV−1 infection indicating a positive modulation of the DNA repair process. Transcriptomic analysis of *S. sclerotiorum* infected with SsHADV-1 also reveals changes in non-homologous end-joining genes [11].

Genes relating to the cell cycle were investigated because of the fungal genomovirus’ similarity to plant geminiviruses and other CRESS-DNA viruses. Many geminiviruses and nanoviruses induce plant cells to re-enter the endocycle and replicate both viral and plant chromosomal DNA [13,46]. Literature has indicated that geminivirus REP often interacts directly with host cell cycle proteins to push the cell into S-phase to utilize the host machinery for RCA [19,47]. RNA-Seq analysis shows that cyclins, CDKs, and other cell cycle-related proteins such as retinoblastoma-related protein (RBR) show differential expression in *S. sclerotiorum* infected with the virus SlaGemV−1. Whi5 is a negative regulator of the cell cycle characterized in yeast which appears as a substitute of RBR [48], and two potential Whi5-like proteins in *S. sclerotiorum* are both up regulated under SlaGemV−1 infection. The protease separase, necessary for cohesion cleavage and allowing of chromosomal segregation during anaphase [49], is also up regulated under the SlaGemV−1 infection in the cabbage leaf curl virus (CLCV) Begomovirus, a bipartite geminivirus, the endocycle was induced by modifying the expression of cyclin D3 family members, which regulate CDKs during G1 phase and interact with the plant RBR-E2F system to encourage polyploidy and replication of the viral genome without mitosis [44]. Here, we see mostly up regulation of *S. sclerotiorum* cyclins and CDKs. Likely, SlaGemV−1 infection induces the cell cycle similar to other DNA viruses which induce cell proliferation [50] or which induce endocycle replication [44,51,52].

Similarly, *S. sclerotiorum* strain DT-8 which harbors the gemycircularvirus SsHADV-1 also shows differential expression through RNA-Seq analysis which shows similar changes in GO enrichments as seen in SlaGemV−1-infected DK3 [11]. Qu et al. also show similar GO enrichments of DNA replication/repair amongst their up regulated genes as well as GO enrichments of carbohydrate metabolism and binding terms amongst their down regulated pathways.

Ubiquitylation pathways are known to be disturbed by some geminivirus infections [13], with some geminiviruses acting as triggers to induce ubiquitin pathways as defense mechanisms and others utilizing host ubiquitin pathways to their benefit [53]. Ubiquitin-like 1-activating enzyme E1 B is an E1 protein which shows the most differential expression of its class responsible for the initial binding/sequestering of ubiquitin. Two putative ubiquitin-conjugating enzyme E2 Qs and ubiquitin-conjugating enzyme E2 H are of the E2 class of ubiquitination enzymes which are necessary for interaction with ubiquitin conjugation class E3 and polyubiquitination of target proteins. Of these, type H is down regulated while type Q enzymes are up regulated. Putative target recognizing subunit cdc20 is a down regulated E3 protein which binds both the target substrate and the E2 complex for polyubiquitination. Cdc20 also acts with APC/C and together modulate the cell cycle and is involved in chromosome segregation and DNA synthesis pathways. Cdc20 also acts as the ubiquitylation enzyme E3 which polyubiquinates the protein responsible for the inhibition of separase [54], which is also seen up regulated. Major changes in ubiquitination pathways can disrupt proteolysis and cell cycle controls. Differential expression of cyclins and ubiquitin proteolysis pathway components may induce changes in the cell cycle and proliferation of the virus genome.

### 4.3. Silencing, and Methylation

Fungal RNAi pathways rely on Dicers, Argonautes, and RdRps and serve various functions, one of which is defense against viral infection by targeting their RNA genomes/transcribed sequences [55]. Similar to *Sclerotinia sclerotiorum* hypovirus 2-L (SsHV2-L) infection on *S. sclerotiorum*, the expression of some silencing pathway genes is not significantly altered [10]. Table S7 shows that *S. sclerotiorum* dicers 1 and 2 as well as argonaute 2 are not differentially expressed. Argonaute 4 shows slightly increased expression and RdRp genes SAD1 and RRP3 show down regulation. However, confirmation by RT-qPCR reveals that these down regulations are very slight or non-significant. SlaGemV−1 infection may not induce a modulation of host RNAi systems similar to what is seen during SsHADV-1 infection [11].

Similar to plant geminivirus infections, some changes in methylation genes can be observed. Similarly as described by Hanley-Bowdoin, the putative ADK homolog of *S. sclerotiorum* appears down regulated and is necessary for S-adenosyl methionine synthesis, as well homolog putative SAHH protein which interacts with geminiviruses in plants is also down regulated [13]. A DIM2 important for cystine methylation and RNAi [56] sees an up regulation, further DIM2 shares homology to chromomethylase 3, a gene identified to be affected by geminivirus infection [13,57]. Along with the potential down regulation of other methylation genes (Table S8), methylation-dependent transcriptional gene silencing as well as other methylation-dependent gene pathways may be influenced by SlaGemV−1-infection.

### 4.4. Differential Expression of Pathogenesis and Polysaccharide Metabolism-Related Genes in S. sclerotiorum

SlaGemV−1 causes visible changes in the fungal morphology, infection potential, and growth rate of *S. sclerotiorum*, which may be caused by compounding changes in genetic expression seen through RNA-Seq. Here, we have determined that alongside these phenotypes, differences in genetic expression are also readily visible by RNA-Seq and differential expression analysis. Multiple major down regulations are immediately noticed in the differential expression of SlaGemV−1-infected *S. sclerotiorum*: Endo2, SsCP1,SsNep1, SsNep2 and SsSSVP1, of which Endo2 was also seen differentially down regulated by SsHADV-1 by Qu et al. [11].

Significant decreases in Endo2, an endo-β-1,4-glucanase aiding in the saccharification of cellulose [39], and SsSSVP1, a cysteine-rich secreted protein of *S. sclerotiorum* which induces plant cell death through the targeting of plant mitochondrial QCR8, a cytochrome b-c_1_ complex subunit [58], may indicate major reductions in cellulose breakdown and plant cell death induction. SsSsvp1 has also been indicated as a pathogenicity factor in other RNA-Seq studies [59,60,61]. Thioredoxin1 (SsTrx1), an important ROS production protein which has been shown to be necessary for pathogenesis [62], shows down regulation during SlaGemV−1 infection. SsNep2 is a necrosis and ethylene-inducing peptide [59] and triggers hypersensitive responses in plants as shown through agroinfiltration trials [63]. Ss-Sl2 and pph1, genes related to sclerotial development and fungal cell wall integrity, are also down regulated. Pph1 encodes the catalytic subunit of a type A2 Ser/Thr phosphatase (PP2A) which should show normal regulation regardless of infection and when silenced has shown almost complete arrest of hyphal growth and the activation of the protein PP2A [64]. Suppression of pph1 has also been seen as an effect of the antibiotic agent wuyiencin [65]. The PP2A subunit A protein also shows down regulation during infection of SlaGemV−1. Significant reductions in carbohydrate metabolism pathways may also indicate a lowered efficacy of SlaGemV−1-infected *S. sclerotiorum* to break down cellulose.

Cellulose and polysaccharide bindings show enrichment among the down regulated genes in presence of SlaGemV−1 (GO:0030248 and 0030247) as do carbohydrate metabolic and catabolic processes (GO:0005975 and GO:0016052). Differential expression of genes relating to chitin and cellulose metabolism show significant down regulation. Cellulase, glucanases, and glycoside hydrolases all see significant down regulation. We also see changes in the fungal chitin biosynthesis and metabolism with changes in chitinases and chitin biosynthesis (*Sschs*). The chitin biosynthesis gene *Sschs* remains marginally up regulated while down regulation of endochitinase 33 and B are also observed, while endochitinase A remains up regulated. Two putative glycoside hydrolase genes were also seen down regulated. In contrast to a previous study where Marzano et al. found that infection of the hypovirulence-inducing SsHV2-L on *S. sclerotiorum* up regulated genes relating to carbohydrate metabolism [10], here we find that carbohydrate metabolism is instead down regulated by SlaGemV−1 infection. The difference could be due to different growth conditions between the two studies.

Oxaloacetate is the precursor to the important virulence factor for *S. sclerotiorum* oxalic acid, as the GO enrichment for oxoacid metabolism is down regulated, not only may the bioproduction of oxalic acid be reduced, but further products synthesized from oxaloacetate including citric acid may also be affected. The biosynthesis and metabolism of oxaloacetate and oxalic acid may be differentially expressed affecting general metabolism and pathogenesis. Oxalic acid is an important metabolite for virulence potential of *S. sclerotiorum* [42]. *Oah*, responsible for the biosynthesis of oxalic acid from oxaloacetate, remains unchanged during virus infection whereas oxalate decarboxylase 1 and 2 (*odc*1 and *odc*2) are both down regulated in presence of SlaGemV−1 infection. Loss-of-function mutants of *odc*2 hyperaccumulated oxalic acid and were unable to infect plants unless the plants were pre-wounded [66]. *Sod*1, A Cu/Zn superoxide dismutase gene, mutants halve oxalate production [67] and appears down regulated during SlaGemV−1 infection. Formate dehydrogenase is also seen highly down regulated during virus infection which is another gene present in the breakdown of oxalic acid. Further, the GO enrichment of oxoacid metabolic processes (GO:0043436) is significantly changed among the down regulated genes. Two citrate synthase genes see down regulation as does a pyruvate carboxylase and malate dehydrogenase. Acetyl-CoA C-acetyltransferase is an important enzyme for the biosynthesis of citrate in the Krebs cycle metabolic pathway and is seen down regulated. Differential regulation of malate dehydrogenase and pyruvate carboxylase which are important for oxaloacetate biosynthesis via the Krebs cycle may lead to both lower levels of oxalic acid production and may also directly affect the efficiency of this important metabolic process. Quantification of oxalic acid concentration would reveal more about the phenotypic effects that deregulation of these genes has under SlaGemV−1 infection in *S. sclerotiorum*.

### 4.5. Sclerotial Development and Melanization

Several genes relating to sclerotial development which are reviewed by Xu et al. and Xia et al. also show significant changes in *S. sclerotiorum* upon SlaGemV−1 infection [42,43]. The up regulated *Pac*1 gene is a putative transcription factor which regulates genetic expression in response to environmental pH and normally shows expression positively correlated to the pH of its growing medium [68]. *Pac*1 has also been shown necessary for sclerotia development and virulence as signaling regulator for oxalic acid accumulation [69], although the actual oxalic acid producing gene *oah* is not differentially expressed in the current study. *Sop*1 is a microbial opsin connected to sclerotial development and virulence in *S. sclerotiorum* [70].

Melanin biosynthesis genes in *S. sclerotiorum* which are instrumental in sclerotial development and the pathogen’s ability to overwinter are not differentially expressed, but several genes relating to sclerotial formation are [71]. Two melanin biosynthesis genes shown to be important for sclerotial development, *Scd*1 and *Thr*1 [71], do not show significant changes in expression while the melanin biosynthesis gene Pks1 is up regulated, but other genes corresponding to sclerotial development do show other changes in expression. Down regulation of the transcription factor, *smr*1, is observed, can also explain a change in melanization and sclerotial development. Mutants of smr1 show less melanization, smooth sclerotial formation, greater susceptibility to environmental stresses, and a reduction in apothecia formation [72]. Several genes which have been shown to abolish sclerotial formation when deleted show down regulation including *Pth*2 and *sl*2; although *sop*1, an important light-sensitive protein whose deletion abolished sclerotial formation, sees an up regulation. *Sl*2 is a unique Sclerotiniaceae protein which is associated with sclerotia formation, and silenced strains have been shown to greatly lose ability to form melanized, compact sclerotia and sees down regulation [73]. Compounding reductions in the expression of genes which have been shown to affect the size, number, or morphology of sclerotia without total abolition may also help explain the loss of sclerotial formation: *Itl*2, *rgb*1, *Scat*1, and *Fdh*1.

## 5. Conclusions

Gene differential expression analysis reveals significant changes in *S. sclerotiorum* under infection by the hypovirulence-inducing mycovirus SlaGemV−1. Genes relating to the cell cycle and DNA replication/repair pathways are up regulated, specifically homologous end-joining genes. GO enrichments also indicate enrichment of cell-cycle-related genes within the up regulated genes but not within the down regulated genes. Consistent with the GO enrichment in cell membrane genes, TEM imaging suggests SlaGemV−1 particles localize to the cell membrane. Analysis of methylation pathways indicates that SAM-dependent defense-related methylation may be down regulated as seen by the down regulation of ADK. While oxalic acid biosynthesis appears to not be affected during SlaGemV−1 infection, oxaloacetate metabolism appears to be affected, and oxalic acid decarboxylase shows down regulation.

## Data Availability

RNA-Seq.

## Supplementary Materials

The following supporting information can be downloaded at: https://www.mdpi.com/article/10.3390/v14091892/s1, Table S1. Results from raw RNA-Seq libraries after trimming, aligning to the *S. sclerotiorum* genome, and count annotation by subread. Less than 0.01% of reads for each library were removed after trimming. 65.61–72.91% of all raw reads were aligned to the *S. sclerotiorum* genome by HISAT2 while 49.4–74.5% of those genes were correctly aligned to the *S. sclerotiorum* annotation file. Genome and annotation files were both acquired from the JGI MycoCosm database (45); Table S2. GO Molecular functions enrichments calculated by FungiDB. Up and down regulated genes were separated into individual lists before entering into FungiDB search strategies. A larger number of enriched GO IDs were characterized with down regulated genes. DNA-related up regulated genes and polysaccharide/carbohydrate binding down regulated genes would be further investigated for their roles in cell cycle progression, viral lifecycle, and loss of pathogenicity. Cutoff for FDR and Bonferroni adjustments were chosen as *p* ≤ 5.00 × 10^−2^; Table S3. GO Biological processes enrichments calculated by FungiDB. Up and down regulated genes were separated into individual lists before entering into FungiDB search strategies. Similar to the GO Molecular functions search, a larger number of enriched GO IDs were characterized with down regulated genes. Of interest, the up regulated genes relating to the cell cycle and DNA replication/repair would be investigated alongside the down regulated genes relating to small molecule metabolic processes and oxoacid metabolism for their potential roles in oxalic acid biosynthesis and metabolism. Cutoff for FDR and Bonferroni adjustments were chosen as *p* ≤ 5.00 × 10^−2^; Table S4. GO Cellular component enrichments calculated by FungiDB. Up and down regulated genes were separated into individual lists before entering into FungiDB search strategies. Among the up regulated genes, enrichments that are related to chromosomal regions seem to be enriched. Cutoff for FDR and Bonferroni adjustments were chosen as *p* ≤ 5.00 × 10^−2^; Table S5. Differentially expressed proteins related to DNA replication, repair, and the cell cycle. Annotation was determined via KEGG pathway annotations. FDR adjustment cutoff *p* ≤ 5.00 × 10^−2^ used for determining significance; Table S6. Select differentially expressed genes related to ubiquitin proteolysis pathways as determined by KEGG pathway annotation. FDR adjustment cutoff *p* ≤ 5.00 × 10^−2^ used for determining significance; Table S7. Gene silencing pathway expression including known dicers, argonautes, and RdRps. Most differential expression FDR adjustment cutoff *p* ≤ 5.00 × 10^−2^ used for determining significance; Table S8. Methylation-related genes. FDR adjustment cutoff *p* ≤ 5.00 × 10^−2^ used for determining significance; Table S9. pathogenesis-determinate genes. FDR adjustment cutoff *p* ≤ 5.00 × 10^−2^ used for determining significance; Table S10. Differential expression of genes necessary for normal sclerotial development. Melanin biosynthesis genes Scd1 and Thr1 in particular show no significant differences in regulation. Other genes related to sclerotial formation follow a similar trend of down regulation, with the exception of sop1. FDR adjustment cutoff *p* ≤ 5.00 × 10^−2^ used for determining significance; Table S11. Differentially expressed genes relating to carbohydrate metabolism. SlaGemV−1 infection leads to differential expression seen in chitinase genes important to fungal cell wall development as well as in cellulases and glycoside hydrolases used for breaking down and penetrating cell walls. Other carbohydrate genes necessary for normal metabolism are also seen differentially regulated. FDR adjustment cutoff *p* ≤ 5.00 × 10^−2^ used for determining significance; Table S12. Oxalic acid biosynthesis gene *oah* does not show differential expression, but accumulation-related genes odc1&2 as well as metabolism related genes do show differential expression. Oxaloacetate biosynthesis as well as its acetylation into citrate also seem to be down regulated, indicating potentially less accumulation of oxalic acid despite no change in *oah* genes. FDR adjustment cutoff *p* ≤ 5.00 × 10^−2^ used for determining significance; Table S13. Top and bottom 50 results from DESeq2 analysis based on log2 change and a BH-calculated FDR padj ≤ 0.05; Table S14. RT-qPCR primer design; Table S15. RT-qPCR results; Figure S1. Three mean normalization methods included with DESeq2 for the differential expression of *S. sclerotiorum*. From left to right: normal transformation, regularized log transformation, and variance stabilization were all tested on the whole dataset. Variance stabilization was used throughout the analysis as it provided the flattest moving average line indicated in red; Figure S2. A second differential expression toolset was used through R called edgeR. After analysis, a Venn diagram showing unique and shared differentially expressed genes in SlaGemV−1-infected DK3 determined by FDR ≤ 0.05 and LFC ≤ −0.5 or ≥0.5 using datasets determined through both DESeq2 and edgeR algorithms were compared. Further data analysis was proceeded with the DESeq2 dataset, the more stringent of the two methods; Figure S3. Distance matrix visualized by a heatmap of the differential expression of all *S. sclerotiorum* cell cycle-related genes as annotated by KEGG.
