# Supplementary material for: Characterization of Transcriptional Responses to Genomovirus Infection of the White Mold Fungus, Sclerotinia sclerotiorum
Source: Viruses. 2022 Aug 27;14(9):1892. doi: 10.3390/v14091892 (PMC9506476; doi:10.3390/v14091892)
Supplement: Supplementary file 1 [file viruses-14-01892-s001.zip › viruses-1808337-supplementary.pdf]

Supplemental Data

Table S1. Results from raw RNA-Seq libraries after trimming, aligning to the *S. sclerotiorum* genome, and count annotation by subread. Less than 0.01% of reads for each library were removed after trimming. 65.61%-72.91% of all raw reads were aligned to the *S. sclerotiorum* genome by HISAT2 while 49.4%-74.5% of those genes were correctly aligned to the *S. sclerotiorum* annotation file. Genome and annotation files were both acquired from the JGI MycoCosm database (45).

Library	Counts	Trimmed Counts	Percent trimmed	HISAT2 Alignment counts	HISAT2 Alignment %	Subread assignment counts	Subread assignment %
**DK3_1_1**	26,379,991	26,379,211	2.96E-03	26,379,211	72.91	37,435,058	49.4
**DK3_1_2**	26,379,991	26,379,211					
**DK3_2_1**	24,855,157	24,854,390	3.09E-03	24,854,390	68.59	41,296,229	74.9
**DK3_2_2**	24,855,157	24,854,390					
**DK3_3_1**	25,929,565	25,928,732	3.21E-03	25,928,732	70.83	39,568,260	59.4
**DK3_3_2**	25,929,565	25,928,732					
**DK3_4_1**	24,875,286	24,874,424	3.47E-03	24,874,424	69.09	39,860,413	69.0
**DK3_4_2**	24,875,286	24,874,424					
**DK3V_1_1**	24,674,643	24,673,536	4.49E-03	24,673,536	66.56	38,609,304	70.9
**DK3V_1_2**	24,674,643	24,673,536					
**DK3V_2_1**	22,661,345	22,660,729	2.72E-03	22,660,729	67.77	35,380,663	67.8
**DK3V_2_2**	22,661,345	22,660,729					
**DK3V_3_1**	22,070,687	22,069,659	4.66E-03	22,069,659	65.61	33,641,762	73.0
**DK3V_3_2**	22,070,687	22,069,659					
**DK3V_4_1**	22,093,719	22,092,965	3.41E-03	22,092,965	66.35	33,869,492	72.2
**DK3V_4_2**	22,093,719	22,092,965					

Table S2. GO Molecular functions enrichments calculated by FungiDB. Up and downregulated genes were separated into individual lists before entering into FungiDB search strategies. A larger number of enriched GO IDs were characterized with downregulated genes. DNA-related upregulated genes and polysaccharide/carbohydrate binding downregulated genes would be further investigated for their roles in cell cycle progression, viral lifecycle, and loss of pathogenicity. Cutoff for FDR & Bonferroni adjustments were chosen as P ≤ 5.00E-2.

ID	Name	Bgd	count	% bgd	Fold enrichment	Odds	P	FDR	Bonferroni	Reg.
**GO:0140097**	catalytic activity, acting on DNA	105	52	49.5	2.08	3.21	7.26E-09	7.35E-06	7.35E-06	UP
**GO:0003677**	DNA binding	539	181	33.6	1.41	1.69	5.78E-08	2.92E-05	5.85E-05	UP
**GO:0008094**	ATP-dependent activity, acting on DNA	36	23	63.9	2.68	5.73	3.43E-07	0.000116	0.000348	UP
**GO:0043167**	ion binding	1895	517	27.3	1.15	1.29	2.39E-05	0.00573	0.024233	UP
**GO:0003678**	DNA helicase activity	23	15	65.2	2.74	6.05	2.83E-05	0.00573	0.028651	UP

ID	Name	Bgd	count	% bgd	Fold enrichment	Odds	P	FDR	Bonferroni	Reg.
**GO:0003824**	catalytic activity	3133	927	29.6	1.17	1.48	2.56E-13	3.09E-10	3.09E-10	DOWN
**GO:0030248**	cellulose binding	24	21	87.5	3.46	20.88	2.42E-10	1.46E-07	2.91E-07	DOWN
**GO:0030247**	polysaccharide binding	28	23	82.1	3.24	13.73	4.26E-10	1.71E-07	5.13E-07	DOWN
**GO:0008135**	translation factor activity, RNA binding	53	32	60.4	2.39	4.56	6.28E-08	1.77E-05	7.57E-05	DOWN
**GO:0003743**	translation initiation factor activity	37	25	67.6	2.67	6.22	7.34E-08	1.77E-05	8.85E-05	DOWN
**GO:0090079**	translation regulator activity, nucleic acid binding	54	32	59.3	2.34	4.35	1.17E-07	2.35E-05	0.000141	DOWN
**GO:0016829**	lyase activity	133	61	45.9	1.81	2.55	1.78E-07	3.07E-05	0.000215	DOWN
**GO:0045182**	translation regulator activity	56	32	57.1	2.26	3.99	3.76E-07	5.67E-05	0.000453	DOWN
**GO:0030246**	carbohydrate binding	60	32	53.3	2.11	3.41	2.97E-06	0.000399	0.003588	DOWN
**GO:0016830**	carbon-carbon lyase activity	46	26	56.5	2.23	3.88	6.18E-06	0.000746	0.007457	DOWN
**GO:0016667**	oxidoreductase activity, acting on a sulfur group of donors	28	18	64.3	2.54	5.35	1.50E-05	0.001643	0.018074	DOWN
**GO:0016491**	oxidoreductase activity	727	230	31.6	1.25	1.42	3.11E-05	0.003122	0.037465	DOWN

Table S3. GO Biological processes enrichments calculated by FungiDB. Up and downregulated genes were separated into individual lists before entering into FungiDB search strategies. Similar to the GO Molecular functions search, a larger number of enriched GO IDs were characterized with downregulated genes. Of interest, the upregulated genes relating to the cell cycle and DNA replication/repair would be investigated alongside the downregulated genes relating to small molecule metabolic processes and oxoacid metabolism for their potential roles in oxalate biosynthesis and metabolism. Cutoff for FDR & Bonferroni adjustments were chosen as $P \leq 5.00E-2$.

ID	Name	Bgd	count	% bgd	Fold enrichment	Odds	P	FDR	Bonferroni	Reg.
**GO:0007049**	cell cycle	153	79	51.6	2.17	3.53	4.89E-14	5.64E-11	1.05E-10	UP
**GO:0051276**	chromosome organization	121	67	55.4	2.33	4.09	5.25E-14	5.64E-11	1.13E-10	UP
**GO:0006259**	DNA metabolic process	248	106	42.7	1.8	2.48	1.71E-11	1.23E-08	3.68E-08	UP
**GO:0006310**	DNA recombination	61	39	63.9	2.69	5.78	2.62E-11	1.41E-08	5.63E-08	UP
**GO:0000278**	mitotic cell cycle	84	48	57.1	2.4	4.36	4.62E-11	1.99E-08	9.94E-08	UP
**GO:0022402**	cell cycle process	123	62	50.4	2.12	3.34	1.01E-10	3.62E-08	2.17E-07	UP
**GO:1903047**	mitotic cell cycle process	77	44	57.1	2.4	4.35	2.99E-10	9.19E-08	6.43E-07	UP
**GO:0006974**	response to DNA damage stimulus	170	77	45.3	1.9	2.73	4.26E-10	1.14E-07	9.14E-07	UP
**GO:0006281**	DNA repair	152	70	46.1	1.94	2.81	1.10E-09	2.64E-07	2.37E-06	UP
**GO:0006260**	DNA replication	83	45	54.2	2.28	3.87	1.92E-09	4.12E-07	4.12E-06	UP
**GO:0000280**	nuclear division	52	31	59.6	2.5	4.79	3.31E-08	6.47E-06	7.11E-05	UP
**GO:0006261**	DNA-dependent DNA replication	39	25	64.1	2.69	5.79	9.53E-08	1.71E-05	0.000205	UP
**GO:0000003**	reproduction	55	31	56.4	2.37	4.19	1.99E-07	3.13E-05	0.000427	UP
**GO:0071103**	DNA conformation change	33	22	66.7	2.8	6.47	2.04E-07	3.13E-05	0.000439	UP
**GO:0051716**	cellular response to stimulus	414	142	34.3	1.44	1.73	4.37E-07	6.26E-05	0.000939	UP

ID	Name	Bgd	count	% bgd	Fold enrichment	Odds	P	FDR	Bonferroni	Reg.
**GO:0033554**	cellular response to stress	260	96	36.9	1.55	1.93	9.55E-07	0.000128	0.002052	UP
**GO:0051321**	meiotic cell cycle	38	23	60.5	2.54	4.96	1.38E-06	0.000166	0.002972	UP
**GO:0006302**	double-strand break repair	43	25	58.1	2.44	4.5	1.39E-06	0.000166	0.002979	UP
**GO:0048285**	organelle fission	59	31	52.5	2.21	3.59	1.58E-06	0.000171	0.003404	UP
**GO:0022414**	reproductive process	51	28	54.9	2.31	3.95	1.60E-06	0.000171	0.003429	UP
**GO:0000724**	homologous recombination	27	18	66.7	2.8	6.46	2.71E-06	0.000278	0.00583	UP
**GO:1903046**	meiotic cell cycle process	32	20	62.5	2.63	5.39	3.37E-06	0.00032	0.007238	UP
**GO:0050896**	response to stimulus	449	148	33	1.39	1.63	3.43E-06	0.00032	0.007361	UP
**GO:0007059**	chromosome segregation	58	30	51.7	2.17	3.47	3.57E-06	0.00032	0.007674	UP
**GO:0010564**	regulation of cell cycle process	61	31	50.8	2.14	3.35	3.99E-06	0.000343	0.008584	UP
**GO:0006270**	DNA replication initiation	15	12	80	3.36	12.89	6.97E-06	0.000576	0.014987	UP
**GO:0000819**	sister chromatid segregation	26	17	65.4	2.75	6.1	7.72E-06	0.000604	0.016582	UP
**GO:0006950**	response to stress	285	100	35.1	1.47	1.78	7.87E-06	0.000604	0.016921	UP
**GO:1901987**	regulation of cell cycle phase transition	49	26	53.1	2.23	3.66	8.69E-06	0.000644	0.018679	UP
**GO:0051301**	cell division	55	28	50.9	2.14	3.36	1.12E-05	0.0008	0.024009	UP
**GO:0051726**	regulation of cell cycle	78	36	46.2	1.94	2.78	1.17E-05	0.000813	0.025194	UP
**GO:0000725**	recombinational repair	29	18	62.1	2.61	5.28	1.21E-05	0.000814	0.026044	UP
**GO:0006298**	mismatch repair	12	10	83.3	3.5	16.1	2.32E-05	0.001513	0.049932	UP
**GO:0044281**	small molecule metabolic process	582	237	40.7	1.61	2.18	1.31E-17	3.02E-14	3.02E-14	DOWN
**GO:0009056**	catabolic process	547	220	40.2	1.59	2.12	1.19E-15	1.38E-12	2.76E-12	DOWN
**GO:0005975**	carbohydrate metabolic process	298	134	45	1.78	2.52	4.39E-14	3.38E-11	1.01E-10	DOWN
**GO:0043436**	oxoacid metabolic process	317	140	44.2	1.74	2.44	6.66E-14	3.85E-11	1.54E-10	DOWN
**GO:0006082**	organic acid metabolic process	321	141	43.9	1.74	2.42	9.11E-14	4.21E-11	2.11E-10	DOWN
**GO:0019752**	carboxylic acid metabolic process	311	137	44.1	1.74	2.43	1.60E-13	6.16E-11	3.70E-10	DOWN
**GO:1901575**	organic substance catabolic process	493	195	39.6	1.56	2.04	4.15E-13	1.37E-10	9.59E-10	DOWN
**GO:0044248**	cellular catabolic process	408	166	40.7	1.61	2.12	1.75E-12	4.58E-10	4.05E-09	DOWN
**GO:1901564**	organonitrogen compound metabolic process	1326	438	33	1.3	1.6	1.78E-12	4.58E-10	4.12E-09	DOWN
**GO:0008152**	metabolic process	3212	933	29	1.15	1.42	5.57E-11	1.29E-08	1.29E-07	DOWN
**GO:0006091**	generation of precursor metabolites and energy	86	50	58.1	2.3	4.18	8.46E-11	1.78E-08	1.95E-07	DOWN
**GO:0008150**	biological process	4363	1215	27.8	1.1	1.4	6.76E-10	1.30E-07	1.56E-06	DOWN
**GO:1901566**	organonitrogen compound biosynthetic process	597	215	36	1.42	1.75	8.87E-10	1.58E-07	2.05E-06	DOWN
**GO:0043603**	cellular amide metabolic process	300	121	40.3	1.59	2.06	4.02E-09	6.64E-07	9.30E-06	DOWN
**GO:0044262**	cellular carbohydrate metabolic process	76	43	56.6	2.24	3.91	5.67E-09	8.74E-07	1.31E-05	DOWN
**GO:0044237**	cellular metabolic process	2383	703	29.5	1.17	1.38	8.11E-09	1.17E-06	1.87E-05	DOWN
**GO:0009987**	cellular process	3655	1030	28.2	1.11	1.35	1.03E-08	1.40E-06	2.37E-05	DOWN
**GO:0016052**	carbohydrate catabolic process	134	64	47.8	1.89	2.76	1.28E-08	1.65E-06	2.97E-05	DOWN
**GO:0002181**	cytoplasmic translation	33	24	72.7	2.87	7.96	1.39E-08	1.70E-06	3.22E-05	DOWN
**GO:0006518**	peptide metabolic process	247	102	41.3	1.63	2.14	1.65E-08	1.90E-06	3.80E-05	DOWN
**GO:0043043**	peptide biosynthetic process	229	96	41.9	1.66	2.19	1.79E-08	1.95E-06	4.14E-05	DOWN
**GO:0019637**	organophosphate metabolic process	266	108	40.6	1.6	2.08	1.86E-08	1.95E-06	4.29E-05	DOWN

ID	Name	Bgd	count	% bgd	Fold enrichment	Odds	P	FDR	Bonferroni	Reg.
**GO:0019693**	ribose phosphate metabolic process	94	48	51.1	2.02	3.13	6.35E-08	6.38E-06	0.000147	DOWN
**GO:0006412**	translation	222	92	41.4	1.64	2.14	6.90E-08	6.64E-06	0.000159	DOWN
**GO:0046034**	ATP metabolic process	52	31	59.6	2.36	4.41	1.52E-07	1.41E-05	0.000352	DOWN
**GO:0006163**	purine nucleotide metabolic process	83	43	51.8	2.05	3.22	1.81E-07	1.61E-05	0.000419	DOWN
**GO:0043604**	amide biosynthetic process	255	101	39.6	1.56	1.99	2.32E-07	1.99E-05	0.000536	DOWN
**GO:0009150**	purine ribonucleotide metabolic process	79	41	51.9	2.05	3.23	3.28E-07	2.71E-05	0.000758	DOWN
**GO:0071704**	organic substance metabolic process	2590	745	28.8	1.14	1.32	3.41E-07	2.72E-05	0.000789	DOWN
**GO:0044282**	small molecule catabolic process	101	49	48.5	1.92	2.83	3.58E-07	2.76E-05	0.000828	DOWN
**GO:0009168**	purine ribonucleoside monophosphate biosynthetic process	18	15	83.3	3.29	14.86	3.92E-07	2.92E-05	0.000907	DOWN
**GO:1901565**	organonitrogen compound catabolic process	245	97	39.6	1.56	1.99	4.13E-07	2.98E-05	0.000954	DOWN
**GO:0006520**	cellular amino acid metabolic process	205	84	41	1.62	2.1	4.59E-07	3.21E-05	0.001061	DOWN
**GO:0009259**	ribonucleotide metabolic process	89	44	49.4	1.95	2.93	7.27E-07	4.94E-05	0.001681	DOWN
**GO:0006413**	translational initiation	31	21	67.7	2.68	6.26	7.84E-07	5.18E-05	0.001813	DOWN
**GO:0044271**	cellular nitrogen compound biosynthetic process	602	203	33.7	1.33	1.56	9.13E-07	5.86E-05	0.00211	DOWN
**GO:0072521**	purine-containing compound metabolic process	98	47	48	1.89	2.76	9.45E-07	5.90E-05	0.002184	DOWN
**GO:0009127**	purine nucleoside monophosphate biosynthetic process	17	14	82.4	3.25	13.87	1.30E-06	7.93E-05	0.003013	DOWN
**GO:0044275**	cellular carbohydrate catabolic process	32	21	65.6	2.59	5.69	1.74E-06	0.000103	0.004018	DOWN
**GO:0005976**	polysaccharide metabolic process	106	49	46.2	1.83	2.58	2.16E-06	0.000125	0.005002	DOWN
**GO:0072522**	purine-containing compound biosynthetic process	57	31	54.4	2.15	3.56	2.45E-06	0.000138	0.005661	DOWN
**GO:0006096**	glycolytic process	14	12	85.7	3.39	17.81	3.62E-06	0.000187	0.008354	DOWN
**GO:0006757**	ATP generation from ADP	14	12	85.7	3.39	17.81	3.62E-06	0.000187	0.008354	DOWN
**GO:0044247**	cellular polysaccharide catabolic process	14	12	85.7	3.39	17.81	3.62E-06	0.000187	0.008354	DOWN
**GO:0016051**	carbohydrate biosynthetic process	33	21	63.6	2.51	5.21	3.64E-06	0.000187	0.00842	DOWN
**GO:0009179**	purine ribonucleoside diphosphate metabolic process	16	13	81.3	3.21	12.87	4.28E-06	0.000206	0.009898	DOWN
**GO:0046031**	ADP metabolic process	16	13	81.3	3.21	12.87	4.28E-06	0.000206	0.009898	DOWN
**GO:0009135**	purine nucleoside diphosphate metabolic process	16	13	81.3	3.21	12.87	4.28E-06	0.000206	0.009898	DOWN
**GO:0009126**	purine nucleoside monophosphate metabolic process	20	15	75	2.96	8.92	4.36E-06	0.000206	0.010081	DOWN
**GO:0046390**	ribose phosphate biosynthetic process	64	33	51.6	2.04	3.18	5.53E-06	0.000256	0.012788	DOWN
**GO:0009156**	ribonucleoside monophosphate biosynthetic process	27	18	66.7	2.63	5.95	7.01E-06	0.000318	0.016196	DOWN
**GO:0055086**	nucleobase-containing small molecule metabolic process	183	73	39.9	1.58	2	8.19E-06	0.000358	0.018938	DOWN
**GO:0044264**	cellular polysaccharide metabolic process	44	25	56.8	2.24	3.92	8.22E-06	0.000358	0.018996	DOWN
**GO:0051156**	glucose 6-phosphate metabolic process	11	10	90.9	3.59	29.66	9.00E-06	0.000385	0.020789	DOWN
**GO:0006164**	purine nucleotide biosynthetic process	52	28	53.8	2.13	3.48	9.69E-06	0.000407	0.022392	DOWN
**GO:0006793**	phosphorus metabolic process	465	158	34	1.34	1.57	1.01E-05	0.000415	0.023239	DOWN
**GO:0009117**	nucleotide metabolic process	135	57	42.2	1.67	2.19	1.11E-05	0.00045	0.025635	DOWN
**GO:0009161**	ribonucleoside monophosphate metabolic process	30	19	63.3	2.5	5.14	1.19E-05	0.000468	0.027412	DOWN
**GO:0030243**	cellulose metabolic process	13	11	84.6	3.34	16.32	1.23E-05	0.000468	0.028529	DOWN
**GO:0051275**	beta-glucan catabolic process	13	11	84.6	3.34	16.32	1.23E-05	0.000468	0.028529	DOWN
**GO:0030245**	cellulose catabolic process	13	11	84.6	3.34	16.32	1.23E-05	0.000468	0.028529	DOWN
**GO:0046939**	nucleotide phosphorylation	15	12	80	3.16	11.87	1.39E-05	0.000504	0.032091	DOWN

ID	Name	Bgd	count	% bgd	Fold enrichment	Odds	P	FDR	Bonferroni	Reg.
**GO:0006165**	nucleoside diphosphate phosphorylation	15	12	80	3.16	11.87	1.39E-05	0.000504	0.032091	DOWN
**GO:0002183**	cytoplasmic translational initiation	17	13	76.5	3.02	9.65	1.40E-05	0.000504	0.032274	DOWN
**GO:0006753**	nucleoside phosphate metabolic process	136	57	41.9	1.66	2.16	1.45E-05	0.000514	0.033429	DOWN

Table S4. GO Cellular component enrichments calculated by fungiDB. Up and downregulated genes were separated into individual lists before entering into FungiDB search strategies. Among the upregulated genes, enrichments which are related to chromosomal regions seem to be enriched. Cutoff for FDR & Bonferroni adjustments were chosen as P ≤ 5.00E-2

ID	Name	Bgd	count	% bgd	Fold	Odds	P	FDR	Bonferroni	Reg.
**GO:0005694**	chromosome	189	96	50.8	2.13	3.44	3.44E-16	1.85E-13	1.85E-13	UP
**GO:0000793**	condensed chromosome	50	29	58	2.44	4.48	2.13E-07	5.72E-05	1.14E-04	UP
**GO:0098687**	chromosomal region	67	33	49.3	2.07	3.15	4.75E-06	6.90E-04	2.56E-03	UP
**GO:0044464**	obsolete cell part	1097	320	29.2	1.23	1.39	5.13E-06	6.90E-04	2.76E-03	UP
**GO:0032300**	mismatch repair complex	7	7	100	4.2	inf	4.28E-05	4.19E-03	2.31E-02	UP
**GO:0005657**	replication fork	19	13	68.4	2.87	6.98	4.68E-05	4.19E-03	2.52E-02	UP
**GO:0016021**	integral component of membrane	2000	537	26.9	1.13	1.25	1.09E-04	6.53E-03	5.85E-02	UP
**GO:0000775**	chromosome, centromeric region	41	21	51.2	2.15	3.39	1.25E-04	6.53E-03	6.72E-02	UP
**GO:0031224**	intrinsic component of membrane	2015	540	26.8	1.13	1.25	1.29E-04	6.53E-03	6.92E-02	UP
**GO:0000779**	condensed chromosome, centromeric region	33	18	54.5	2.29	3.87	1.34E-04	6.53E-03	7.18E-02	UP
**GO:0000776**	kinetochore	33	18	54.5	2.29	3.87	1.34E-04	6.53E-03	7.18E-02	UP
**GO:0000790**	nuclear chromatin	28	16	57.1	2.4	4.3	1.52E-04	6.82E-03	8.18E-02	UP
**GO:0000228**	nuclear chromosome	57	26	45.6	1.92	2.71	2.39E-04	9.89E-03	1.29E-01	UP
**GO:0035861**	site of double-strand break	9	7	77.8	3.27	11.25	9.70E-04	3.48E-02	5.22E-01	UP
**GO:0090734**	site of DNA damage	9	7	77.8	3.27	11.25	9.70E-04	3.48E-02	5.22E-01	UP
**GO:0005737**	cytoplasm	1791	608	33.9	1.34	1.77	1.87E-21	9.54E-19	9.54E-19	DOWN
**GO:0044424**	obsolete intracellular part	610	248	40.7	1.61	2.18	2.58E-18	6.57E-16	1.31E-15	DOWN
**GO:0005829**	cytosol	289	136	47.1	1.86	2.75	2.96E-16	5.04E-14	1.51E-13	DOWN
**GO:0044444**	obsolete cytoplasmic part	545	191	35	1.38	1.66	9.22E-08	1.18E-05	4.70E-05	DOWN
**GO:0031975**	envelope	228	84	36.8	1.46	1.76	5.98E-05	5.08E-03	3.05E-02	DOWN
**GO:0031967**	organelle envelope	228	84	36.8	1.46	1.76	5.98E-05	5.08E-03	3.05E-02	DOWN
**GO:0016282**	eukaryotic 43S preinitiation complex	15	11	73.3	2.9	8.16	1.28E-04	6.46E-03	6.54E-02	DOWN
**GO:0005753**	mitochondrial proton-transporting ATP synthase complex	11	9	81.8	3.23	13.34	1.38E-04	6.46E-03	7.06E-02	DOWN
**GO:0005852**	eukaryotic translation initiation factor 3 complex	13	10	76.9	3.04	9.88	1.39E-04	6.46E-03	7.10E-02	DOWN

ID	Name	Bgd	count	% bgd	Fold	Odds	P	FDR	Bonferroni	Reg.
**GO:0033290**	eukaryotic 48S preinitiation complex	13	10	76.9	3.04	9.88	1.39E-04	6.46E-03	7.10E-02	DOWN
**GO:0005740**	mitochondrial envelope	189	70	37	1.46	1.76	2.01E-04	8.54E-03	1.02E-01	DOWN
**GO:0101031**	chaperone complex	6	6	100	3.95	inf	2.62E-04	9.53E-03	1.33E-01	DOWN
**GO:0005832**	chaperonin-containing T-complex	6	6	100	3.95	inf	2.62E-04	9.53E-03	1.33E-01	DOWN
**GO:0005739**	mitochondrion	440	142	32.3	1.27	1.44	4.33E-04	1.47E-02	2.21E-01	DOWN
**GO:0031966**	mitochondrial membrane	176	64	36.4	1.44	1.71	6.56E-04	2.09E-02	3.35E-01	DOWN
**GO:0005576**	extracellular region	129	49	38	1.5	1.83	9.15E-04	2.75E-02	4.67E-01	DOWN
**GO:0045259**	proton-transporting ATP synthase complex	13	9	69.2	2.73	6.67	1.08E-03	3.06E-02	5.51E-01	DOWN
**GO:0098800**	inner mitochondrial membrane protein complex	36	18	50	1.98	2.97	1.21E-03	3.26E-02	6.19E-01	DOWN
**GO:0008541**	proteasome regulatory particle, lid subcomplex	7	6	85.7	3.39	17.76	1.43E-03	3.66E-02	7.32E-01	DOWN
**GO:0005743**	mitochondrial inner membrane	143	52	36.4	1.44	1.71	2.02E-03	4.91E-02	1.00E+00	DOWN

Table S5. Differentially expressed proteins related to DNA replication, repair, and the cell cycle. Annotation was determined via KEGG pathway annotations. FDR adjustment cutoff P ≤ 5.00E-2 used for determining significance.

Gene Name	ID	ID2	Log2 Fold Change	Fold Change	Adjusted P
**TOP3**	SS1G_12655	sscle_06g053170	1.16	2.24	1.06E-04
**Separase**	SS1G_07342	sscle_06g049920	2.38	5.22	1.26E-27
**RNase H2 subunit C**	SS1G_13856	sscle_10g080650	1.66	3.09	2.74E-03
**RAD51**	SS1G_06782	sscle_13g095360	1.01	2.01	6.18E-09
**Putative XRS2**	SS1G_02656	sscle_04g036230	NA	NA	NS
**Putative RPA**	SS1G_08173	sscle_10g075210	1.33	2.51	5.46E-13
**Putative RNase H**	SS1G_02079	sscle_01g003750	-2.92	-7.55	1.11E-03
**Putative RAD54**	SS1G_11894	sscle_12g090630	1.22	2.34	2.88E-10
**Putative RAD54**	SS1G_12550	sscle_06g053890	1.20	2.29	9.18E-08
**Putative RAD52**	SS1G_10638	sscle_09g071050	1.06	2.09	3.75E-04
**Putative RAD50**	SS1G_12239	sscle_05g042050	1.12	2.17	4.23E-09
**Putative PCNA**	SS1G_12686	sscle_02g013610	NA	NA	NS
**Putative MRE11**	SS1G_09567	sscle_15g103930	1.32	2.50	6.24E-10
**Putative Mps1**	SS1G_01433	sscle_01g008890	2.08	4.24	2.08E-22
**Putative DSS1**	SS1G_01104	sscle_01g011380	-0.74	-1.67	7.17E-03
**Putative Cyclin**	SS1G_04347	sscle_02g017440	3.06	8.34	4.18E-22
**Putative Cyclin**	SS1G_11572	sscle_07g060970	1.90	3.73	2.56E-13
**Putative Cyclin**	SS1G_06752	sscle_13g095140	1.48	2.80	1.28E-10
**Putative cdc20**	SS1G_05727	sscle_05g047680	2.47	5.54	2.39E-39
**Mcm7**	SS1G_12751	sscle_02g013150	1.65	3.14	1.02E-14
**Mcm6**	SS1G_13854	sscle_10g080630	1.17	2.24	1.11E-13
**Mcm4**	SS1G_01278	sscle_01g010020	1.00	2.00	2.57E-05

Gene Name	ID	ID2	Log2 Fold Change	Fold Change	Adjusted P
**Mcm3**	SS1G_13330	sscle_03g031590	1.32	2.50	7.78E-08
**Mcm2**	SS1G_01022	sscle_03g022630	1.01	2.01	4.48E-07
**DNA repair protein XRCC4**	SS1G_02074	sscle_01g003790	0.93	1.90	2.23E-03
**DNA Primase subunit**	SS1G_12588	sscle_06g053650	1.40	2.63	7.19E-08
**DNA polymerase**	SS1G_11504	sscle_07g060430	1.05	2.07	3.25E-07
**DNA Pol ε subunit 4**	SS1G_08576	sscle_10g078390	0.69	1.61	6.59E-04
**DNA Pol ε subunit 2**	SS1G_05217	sscle_08g065030	-0.64	-1.55	2.97E-02
**DNA Pol ε subunit 1**	SS1G_11933	sscle_12g090330	2.09	4.26	1.01E-13
**DNA Pol δ3**	SS1G_08296	sscle_10g076110	0.56	1.47	4.48E-03
**DNA Pol δ2**	SS1G_06107	sscle_05g044800	0.85	1.80	2.70E-05
**DNA helicase**	SS1G_06882	sscle_13g096070	2.22	4.65	9.99E-13
**Cyclin-D-interacting**	SS1G_01399	sscle_01g009140	2.55	5.87	2.46E-23
**DNA Pol II subunit 2**	SS1G_05217	sscle_08g065030	-0.64	-1.55	2.97E-02
**Ku80**	SS1G_07128	sscle_06g051630	0.54	1.46	1.38E-02
**DNA Ligase 4**	SS1G_03342	sscle_07g057330	0.50	1.41	4.16E-02
**DNA Pol4**	SS1G_06612	sscle_13g094010	0.85	1.81	1.94E-03
**Rad27**	SS1G_11175	sscle_12g087800	1.14	2.20	3.22E-10
**Whi5-like**	SS1G_07309	sscle_06g050180	1.17	2.25	1.19E-05
**Whi5-like**	SS1G_14043	sscle_10g079150	0.72	1.64	1.02E-02

Table S6. Select differentially expressed genes related to ubiquitin proteolysis pathways as determined by KEGG pathway annotation. FDR adjustment cutoff P ≤ 5.00E-2 used for determining significance.

Gene Name	ID	ID2	Log_2 Fold Change	Fold Change	Adjusted P
**Ubiquitin-activating enzyme E1 B**	SS1G_02376	sscle_04g034130	0.78	1.72	1.47E-03
**Ubiquitin-conjugating enzyme E2 H**	SS1G_02279	sscle_04g033380	-1.41	-2.65	4.11E-08
**Ubiquitin-conjugating enzyme E2 Q**	SS1G_05994	sscle_05g045680	1.04	2.06	1.90E-05
**Ubiquitin-conjugating enzyme E2 Q**	SS1G_05995	sscle_05g045680	0.60	1.51	2.94E-02
**Cdc20**	SS1G_05727	sscle_05g047680	-2.47	-5.54	2.39E-39
**Arrestin C domain-containing**	SS1G_02395	sscle_04g034280	-1.19	-2.27	3.15E-10
**Ubiquitin**	SS1G_01873	sscle_01g005340	-0.95	-1.93	7.00E-04
**Ubiquitin**	SS1G_11035	sscle_12g088930	-0.57	-1.49	1.79E-02
**Ubiquitin-conjugating enzyme E2 G2**	SS1G_01502	sscle_01g008360	-0.99	-1.98	2.62E-06
**Ubiquitin-conjugating enzyme E2 N**	SS1G_05147	sscle_08g064560	-0.98	-1.97	1.83E-06
**E3 ubiquitin-protein ligase RBX1**	SS1G_00027	sscle_03g030230	-0.67	-1.59	1.83E-03
**Skp1**	SS1G_00543	sscle_03g026230	-0.64	-1.56	1.26E-03

Table S7. Gene silencing pathway expression including known dicers, argonautes, and RdRps. Most differential expression FDR adjustment cutoff P ≤ 5.00E-2 used for determining significance.

Gene Name	ID	ID2	Log_2 Fold Change	Fold Change	Adjusted P
**dcl-1**	SS1G_13747	sscle_10g079850	NA	NA	NS
**dcl-2**	SS1G_10369	sscle_16g109590	NA	NA	NS
**ago-2**	SS1G_00334	sscle_03g027950	NA	NA	NS
**ago-4**	SS1G_11723	sscle_12g091970	1.02	2.02	1.69E-04
**SAD1**	SS1G_13161	sscle_02g020610	-0.78	-1.72	4.09E-04
**QDE1**	SS1G_03377	sscle_07g057040	-0.38	-1.30	7.64E-02
**RRP3**	SS1G_09915	sscle_01g002570	-0.60	-1.52	7.54E-03

Table S8. Methylation-related genes. FDR adjustment cutoff P ≤ 5.00E-2 used for determining significance.

Gene Name	ID	ID2	Log_2 Fold Change	Fold Change	Adjusted P
**ADK**	SS1G_00597	sscle_03g025790	-1.79	-3.45	5.72E-32
**SAHH**	SS1G_01191	sscle_01g010680	-1.66	-3.16	1.72E-12
**Putative DIM2**	SS1G_07976	sscle_11g081900	1.80	3.49	1.10E-14
**Trm82**	SS1G_14282	sscle_04g039750	0.75	1.68	9.43E-03
**SAM-Dependent Methyltransferase**	SS1G_05223	sscle_08g065070	1.90	3.73	1.07E-07
**SAM-dependent methyltransferase**	SS1G_03467	sscle_07g056400	2.37	5.16	2.32E-43
**SAM-dependent methyltransferase**	SS1G_10584	sscle_09g071500	-0.48	-1.40	1.17E-02
**Delta(14)-sterol reductase**	SS1G_09177	sscle_15g106990	-2.21	-4.64	2.79E-24
**similar to polyketide synthase**	SS1G_07099	sscle_06g051840	0.45	1.37	4.23E-02
**similar to cobalamin-independent methionine synthase**	SS1G_09237	sscle_15g106510	2.57	5.94	5.25E-10
**21S rRNA (uridine(2791)-2'-O)-methyltransferase MRM2**	SS1G_10919	sscle_09g068880	1.97	3.93	2.26E-21

Table S9. pathogenesis-determinate genes. FDR adjustment cutoff P ≤ 5.00E-2 used for determining significance.

Gene Name	ID	ID2	Log2 Fold Change	Fold Change	Adjusted P
**Endo2**	SS1G_01828	sscle_01g005720	-3.60	-12.13	7.83E-06
**SsNep1**	SS1G_03080	sscle_04g039420	-12.15	-4555.19	6.94E-18
**SsNep2**	SS1G_11912	sscle_12g090490	-2.21	-4.64	2.90E-12
**SsCP1**	SS1G_10096	sscle_16g107670	-2.24	-4.72	1.01E-06
**SsSSVP1**	SS1G_02068	sscle_01g003850	-3.87	-14.66	1.64E-07
**SsTrx1**	SS1G_ 08534	sscle_10g078030	-1.87	-3.64	2.87E-07
**Pph1**	SS1G_08489	sscle_10g077630	-0.76	-1.69	3.53E-04
**Cry1**	SS1G_05163	sscle_08g064670	-1.13	-2.18	2.19E-02
**PP2A subunit A**	SS1G_07119	sscle_06g051720	-0.61	-1.53	3.01E-03

Table S10. Differential expression of genes necessary for normal sclerotial development. Melanin biosynthesis genes Scd1 and Thr1 in particular show no significant differences in regulation. Other genes related to sclerotial formation follow a similar trend of down regulation, with the exception of sop1. FDR adjustment cutoff P ≤ 5.00E-2 used for determining significance.

Gene Name	ID	ID2	Log_2 Fold Change	Fold Change	Adjusted P
**Scd1**	SS1G_13314	sscle_03g031470	NA	NA	NS
**Thr1**	SS1G_13315	sscle_03g031480	NA	NA	NS
**Pks1**	SS1G_13322	sscle_03g031520	3.84	14.35	6.36E-23
**Pth2**	SS1G_13339	sscle_03g031670	-1.00	-2.00	4.12E-07
**CVNH-domain containing secreted protein**	SS1G_02904	sscle_04g038020	-0.93	-1.90	1.30E-03
**sl2**	SS1G_05917	sscle_05g046240	-2.89	-7.43	1.46E-09
**Nox1**	SS1G_05661	sscle_05g048220	-1.02	-2.03	9.55E-05
**sop1**	SS1G_01614	sscle_01g007450	2.47	5.54	1.46E-10
**Itl2**	SS1G_14133	sscle_08g068500	-2.42	-5.36	2.36E-23
**rgb1**	SS1G_07871	sscle_11g082700	-0.55	-1.46	1.08E-04
**Scat1**	SS1G_02784	sscle_04g037170	-0.99	-1.98	4.24E-04
**Fdh1**	SS1G_10096	sscle_16g107670	-2.24	-4.72	1.01E-06
**SMR1**	NA	sscle_12g091490	-1.43	-2.70	7.80E-03

Table S11. Differentially expressed genes relating to carbohydrate metabolism. SlaGemV-1 infection leads to differential expression seen in chitinase genes important to fungal cell wall development as well as in cellulases and glycoside hydrolases used for breaking down and penetrating cell walls. Other carbohydrate genes necessary for normal metabolism are also seen differentially regulated. FDR adjustment cutoff P ≤ 5.00E-2 used for determining significance.

Gene Description	ID	ID2	Log_2 Fold Change	Fold Change	Adjusted P
**Sshxt2**	SS1G_13734	sscle_10g079770	-5.38	-41.69	3.57E-101

Gene Description	ID	ID2	Log_2 Fold Change	Fold Change	Adjusted P
**Sshxt1**	SS1G_04841	sscle_08g062180	-2.57	-5.92	5.93E-10
**beta-fructofuranosidase**	SS1G_07184	sscle_06g051200	-0.69	-1.62	2.54E-02
**Cellulase**	SS1G_01828	sscle_01g005720	-3.59	-12.04	6.53E-06
**Putative Endoglucanase**	SS1G_00892	sscle_03g023650	-7.34	-161.51	4.01E-33
**Putative glycoside hydrolase**	SS1G_02334	sscle_04g033820	-9.34	-648.58	4.19E-29
**Putative Cellobiohydrolase**	SS1G_04945	sscle_08g063000	-1.04	-2.06	4.30E-02
**Endochitinase 33**	SS1G_11212	sscle_12g087520	-2.15	-4.45	4.57E-11
**CBM1 domain-containing protein**	SS1G_01373	sscle_01g009340	-7.29	-156.07	3.45E-29
**Putative carbohydrate esterase**	SS1G_12765	sscle_02g013030	-2.04	-4.13	3.90E-04
**Putative endoglucanase II**	SS1G_00891	sscle_03g023660	-5.76	-54.22	4.78E-13
**Putative glycoside hydrolase**	SS1G_07656	sscle_11g084420	-11.23	-2396.10	1.23E-38
**Mannan endo-1,4-betamannosidase**	SS1G_00746	sscle_03g024710	-7.42	-171.59	4.38e-56
**Endo-1,4-beta-xylanase**	SS1G_03618	sscle_09g074790	-5.24	-37.91	6.18e-50
**Beta-xylanase**	SS1G_12191	sscle_05g041650	-4.99	-31.76	1.50e-47
**putative glucooligosaccharide oxidase**	SS1G_12200	sscle_05g041720	1.85	3.61	1.38E-04
**Sschs**	SS1G_14116	sscle_10g079750	0.59	1.50	6.32E-04
**Endochitinase A**	SS1G_13155	sscle_02g020660	1.67	3.18	4.87E-07
**Endochitinase B1**	SS1G_11700	sscle_07g062010	-3.22	-9.31	4.99E-19
**Endochitinase B1**	SS1G_11304	sscle_12g086860	-2.27	-4.83	9.63E-24

Table S12. Oxalate biosynthesis gene oah does not show differential expression, but accumulation-related genes odc1&2 as well as metabolism related genes do show differential expression. Oxaloacetate biosynthesis as well as its acetylation into citrate also seem to be downregulated, indicating potentially less accumulation of oxalate despite no change in oah. FDR adjustment cutoff $P \leq 5.00E-2$ used for determining significance.

Gene Name	ID	ID2	Log_2 Fold Change	Fold Change	Adjusted P
**Sod1**	SS1G_00699	sscle_03g025030	-1.23	-2.34	5.87E-13
**Pth2**	SS1G_13339	sscle_03g031670	-0.32	-1.24	NS
**Nox1**	SS1G_05661	sscle_05g048220	1.87	3.66	9.15E-07
**PAC1**	SS1G_07355	sscle_06g049830	2.08	4.22	5.47E-09
**Odc2**	SS1G_10796	sscle_09g069850	-3.60	-12.14	1.65E-21
**Oah**	SS1G_08218	sscle_10g075560	0.64	1.55	NS
**Odc1**	SS1G_08814	sscle_14g099710	-4.10	-17.16	2.35E-09
**Fdh**	SS1G_09038	sscle_14g101500	-3.37	-10.30	6.86E-27
**Citrate synthase**	SS1G_01625	sscle_01g007370	-1.48	-2.79	3.20E-13
**Citrate synthase**	SS1G_04899	sscle_08g062630	-1.44	-2.72	1.34E-08
**Pyruvate carboxylase**	SS1G_12839	sscle_02g012470	-1.41	-2.65	2.13E-16
**Malate dehydrogenase**	SS1G_08975	sscle_14g101040	-0.93	-1.91	1.13E-06
**Acetyl-CoA C-acetyltransferase**	SS1G_00464	sscle_03g026900	-1.17	-2.24	1.99E-05
**Acetyl-CoA hydrolase**	SS1G_03975	sscle_09g072050	-0.65	-1.57	5.08E-03

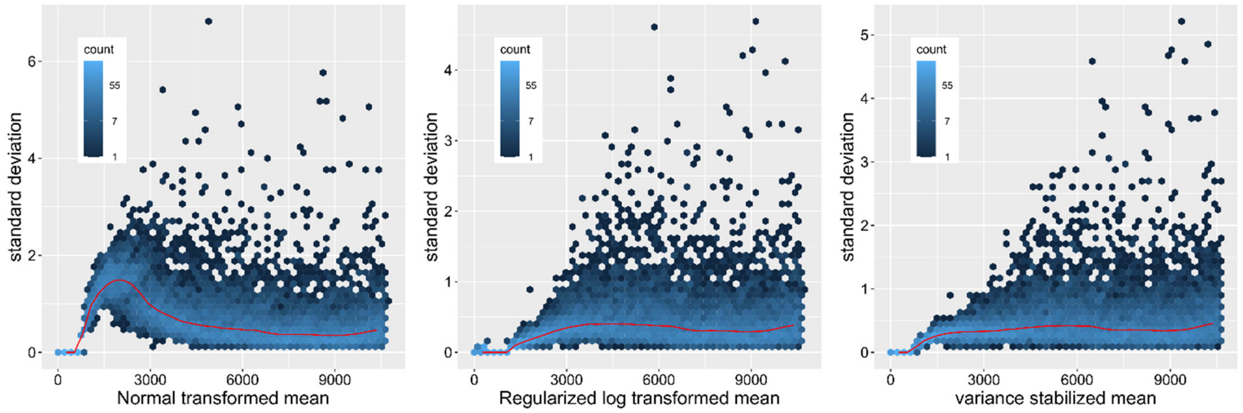

Figure S1. Three mean normalization methods included with DESeq2 for the differential expression of *S. sclerotiorum*. From left to right: normal transformation, regularized log transformation, and variance stabilization were all tested on the whole dataset. Variance stabilization was used throughout the analysis as it provided the flattest moving average line indicated in red.

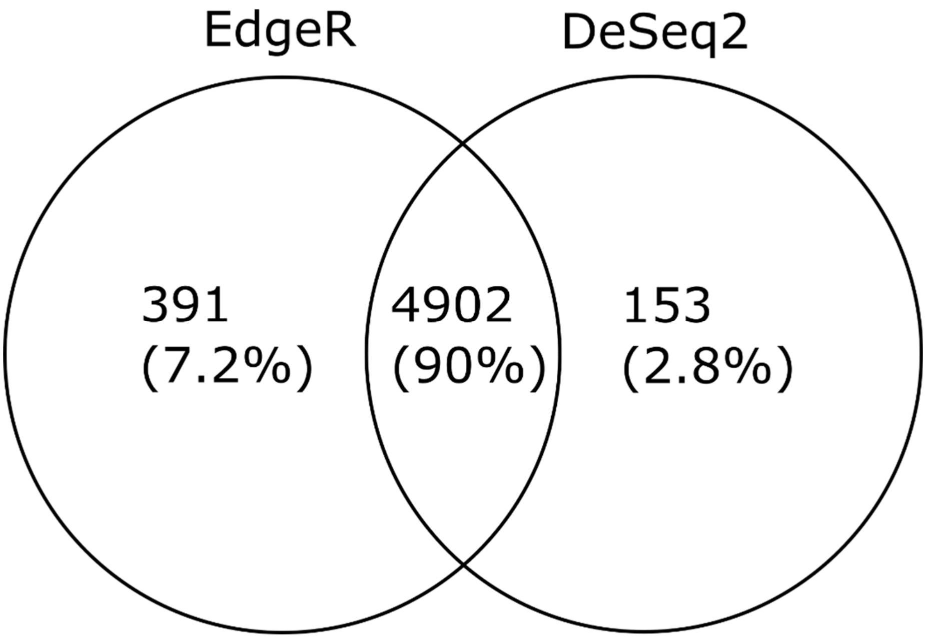

Figure S2. A second differential expression toolset was used through R called edgeR. After analysis, a venn diagram showing unique and shared differentially expressed genes in SlaGemV-1-infected DK3 determined by FDR ≤ 0.05 and LFC ≤ -0.5 or ≥ 0.5 using datasets determined through both DESeq2 and edgeR algorithms were compared. Further data analysis was proceeded with the DESeq2 dataset, the more stringent of the two methods.

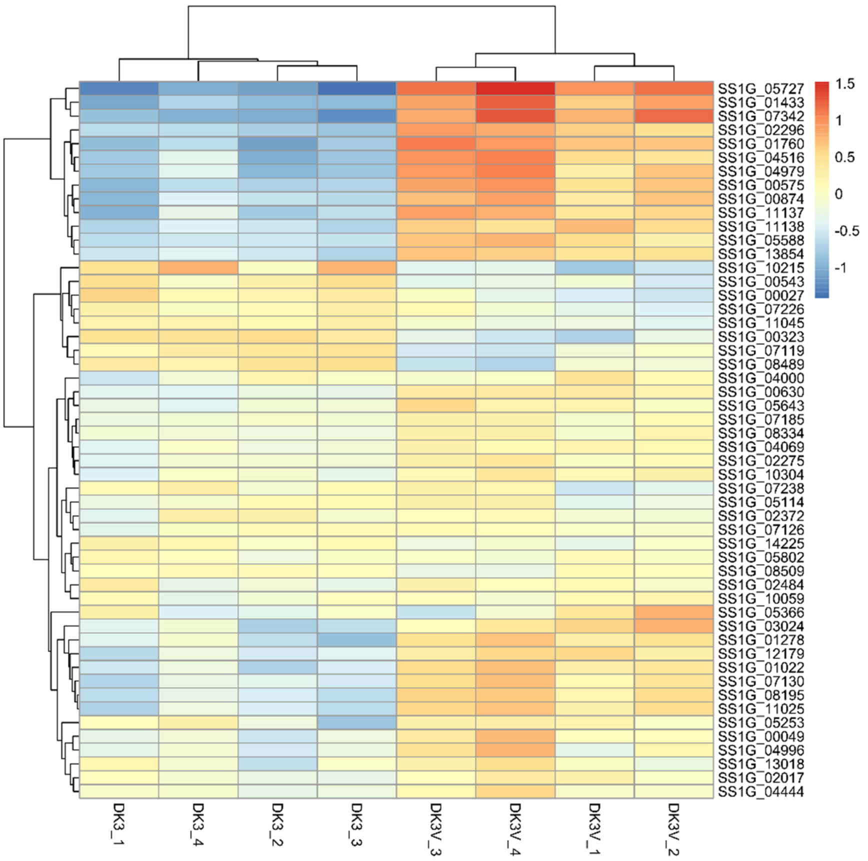

Figure S3. Distance matrix visualized by a heatmap of the differential expression of all *S. sclerotiorum* cell cycle-related genes as annotated by KEGG.

Table S13. Top and bottom 50 results from DESeq2 analysis based on log_2 change and a BH-calculated FDR padj ≤ 0.05.

Gene_ID	Mean	log2 Change	lfcSE	stat	pvalue	padj
SS1G_08398	3711.038	5.371498	0.216966	24.75732	2.59E-135	1.17E-131
SS1G_04213	9495.324	6.106237	0.28391	21.50769	1.32E-102	1.50E-99
SS1G_05765	4932.669	5.993789	0.305674	19.60841	1.31E-85	1.08E-82
SS1G_01102	5356.209	6.456269	0.340718	18.94903	4.50E-80	3.14E-77
SS1G_01019	2956.356	5.463512	0.300049	18.2087	4.40E-74	2.85E-71
SS1G_03683	7536.163	5.475883	0.316157	17.32016	3.31E-67	1.50E-64
SS1G_09578	1117.773	6.172909	0.379701	16.25729	1.98E-59	6.92E-57
SS1G_00702	5025.731	6.173906	0.426242	14.48452	1.52E-47	2.22E-45
SS1G_08201	1062.936	5.886338	0.438071	13.43695	3.67E-41	4.22E-39
SS1G_04598	762.9315	5.666355	0.463664	12.22083	2.41E-34	2.12E-32
SS1G_14215	619.2262	11.9108	1.057393	11.26431	1.97E-29	1.26E-27
SS1G_00703	5957.17	7.166074	0.657359	10.90131	1.14E-27	6.33E-26
SS1G_01100	164.0309	6.158886	0.577266	10.66907	1.42E-26	7.05E-25
SS1G_09248	8780.174	8.825342	0.831672	10.61157	2.63E-26	1.27E-24
SS1G_08203	76.54922	5.966885	0.67567	8.831061	1.04E-18	2.73E-17
SS1G_02694	945.9879	6.393099	0.734948	8.698707	3.36E-18	8.33E-17
SS1G_02695	908.5815	7.494031	0.865197	8.661648	4.65E-18	1.14E-16
SS1G_02828	86234.24	6.250257	0.753428	8.295763	1.08E-16	2.34E-15
SS1G_08202	477.7057	6.360592	0.772938	8.229108	1.89E-16	4.01E-15
SS1G_02692	90.83912	5.2187	0.657945	7.931822	2.16E-15	4.19E-14
SS1G_00506	28.43463	8.187284	1.154114	7.094001	1.30E-12	1.87E-11
SS1G_05762	29.83151	5.781272	0.851314	6.791001	1.11E-11	1.46E-10
SS1G_00704	28.20321	8.175862	1.211776	6.747007	1.51E-11	1.92E-10
SS1G_01654	38.11457	6.143331	0.946128	6.493132	8.41E-11	9.86E-10
SS1G_05833	20.73129	5.671027	0.983363	5.766975	8.07E-09	7.32E-08
SS1G_10484	411.6948	5.494802	0.964444	5.697378	1.22E-08	1.08E-07
SS1G_08163	5116.183	6.166802	1.115177	5.529885	3.20E-08	2.64E-07
SS1G_06089	25.34685	5.530243	1.087374	5.085872	3.66E-07	2.57E-06
SS1G_05014	15.5509	5.863126	1.186087	4.94325	7.68E-07	5.11E-06
SS1G_00508	9.619959	6.623117	1.443186	4.589234	4.45E-06	2.59E-05
SS1G_04972	15.44372	5.856876	1.297115	4.51531	6.32E-06	3.55E-05
SS1G_01532	13.92473	5.694042	1.281086	4.444698	8.80E-06	4.79E-05
SS1G_13766	10.78119	6.049583	1.366782	4.42615	9.59E-06	5.18E-05
SS1G_00385	6.846803	6.133026	1.416262	4.330432	1.49E-05	7.77E-05
SS1G_07042	275.854	5.41447	1.255849	4.311401	1.62E-05	8.44E-05
SS1G_00084	8.31282	5.667959	1.315166	4.30969	1.63E-05	8.50E-05
SS1G_00548	6.976895	6.161504	1.456602	4.230053	2.34E-05	0.000117
SS1G_04374	10.65983	5.284064	1.298363	4.069788	4.71E-05	0.000222
SS1G_06126	5.627239	5.851019	1.516884	3.857261	0.000115	0.000498
SS1G_04191	6.699221	6.100992	1.604716	3.801914	0.000144	0.000615

Gene_ID	Mean	log2 Change	lfcSE	stat	pvalue	padj
SS1G_13505	1269.511	5.39616	1.449054	3.723918	0.000196	0.000812
SS1G_05827	6.303466	6.016585	1.73517	3.467432	0.000525	0.001951
SS1G_00370	4.694226	5.587615	1.635196	3.417091	0.000633	0.002305
SS1G_05299	1437.535	5.923933	1.752756	3.379782	0.000725	0.002611
SS1G_07361	4.817251	5.625087	1.714755	3.280402	0.001037	0.003599
SS1G_13015	5.878855	5.912893	1.90867	3.097912	0.001949	0.006223
SS1G_02579	6.365187	5.277851	1.792754	2.943991	0.00324	0.009736
SS1G_10449	3.872995	5.312292	2.05389	2.586454	0.009697	0.025238
SS1G_10071	8016.02	-8.89786	0.373241	-23.8394	1.30E-125	3.94E-122
SS1G_13754	8955.365	-5.33909	0.241568	-22.1018	3.04E-108	5.52E-105
SS1G_13734	6329.105	-5.38176	0.24808	-21.6936	2.36E-104	3.57E-101
SS1G_01838	42875.05	-9.77365	0.479214	-20.3952	1.85E-92	1.68E-89
SS1G_09251	17649.4	-8.05301	0.462302	-17.4194	5.88E-68	2.97E-65
SS1G_00978	17221.87	-6.60424	0.381187	-17.3254	3.02E-67	1.44E-64
SS1G_03387	17640.23	-7.86727	0.473095	-16.6294	4.27E-62	1.76E-59
SS1G_00977	2284.904	-6.41196	0.389828	-16.4482	8.64E-61	3.27E-58
SS1G_04946	55381.7	-10.0949	0.618324	-16.3263	6.42E-60	2.33E-57
SS1G_00746	36543.67	-7.43301	0.462727	-16.0635	4.60E-58	1.44E-55
SS1G_03618	524.3226	-5.24979	0.344889	-15.2217	2.54E-52	5.62E-50
SS1G_03041	75882.13	-10.8009	0.723401	-14.9307	2.08E-50	3.93E-48
SS1G_00892	55116.81	-7.35529	0.493222	-14.9127	2.72E-50	5.05E-48
SS1G_13872	24262.96	-5.10877	0.34598	-14.7661	2.42E-49	4.07E-47
SS1G_12191	2053.334	-4.90681	0.336431	-14.5849	3.51E-48	5.39E-46
SS1G_01726	6699.306	-6.231	0.433415	-14.3765	7.27E-47	9.85E-45
SS1G_07011	55080.92	-9.00353	0.635974	-14.1571	1.69E-45	2.22E-43
SS1G_10065	7106.423	-5.15398	0.390696	-13.1918	9.78E-40	1.02E-37
SS1G_05582	169568.5	-5.20155	0.420653	-12.3654	4.02E-35	3.65E-33
SS1G_01373	20061.54	-7.30435	0.634985	-11.5032	1.27E-30	8.95E-29
SS1G_02334	130765.5	-9.34975	0.814486	-11.4793	1.68E-30	1.15E-28
SS1G_12118	2595.826	-4.81377	0.428239	-11.2409	2.57E-29	1.62E-27
SS1G_13029	727.4983	-5.14048	0.504362	-10.1921	2.15E-24	8.72E-23
SS1G_03080	11860.34	-12.1533	1.3524	-8.98646	2.55E-19	6.94E-18
SS1G_05552	290.4613	-4.743	0.580401	-8.17195	3.03E-16	6.36E-15
SS1G_00179	138.6642	-4.85128	0.612664	-7.91834	2.41E-15	4.65E-14
SS1G_03909	87.31647	-8.55731	1.122134	-7.62592	2.42E-14	4.21E-13
SS1G_00891	3065.408	-5.77089	0.762258	-7.57078	3.71E-14	6.34E-13
SS1G_13126	4682.824	-4.87108	0.65387	-7.44961	9.36E-14	1.53E-12
SS1G_05007	9158.264	-5.69552	0.797116	-7.14515	8.99E-13	1.32E-11
SS1G_09391	31.1619	-4.71764	0.676558	-6.973	3.10E-12	4.30E-11
SS1G_13141	31.41705	-6.06809	1.105713	-5.48794	4.07E-08	3.30E-07
SS1G_00230	178.5538	-7.12164	1.344079	-5.29853	1.17E-07	8.79E-07
SS1G_11182	13.77447	-4.8589	0.970708	-5.00553	5.57E-07	3.82E-06
SS1G_05416	7.002447	-6.36567	1.400685	-4.54468	5.50E-06	3.14E-05

Gene_ID	Mean	log2 Change	lfcSE	stat	pvalue	padj
SS1G_00806	12.72216	-4.74567	1.307472	-3.62966	0.000284	0.001131
SS1G_02391	5.670803	-5.30982	1.75015	-3.03393	0.002414	0.007525
SS1G_06289	3.174358	-5.22424	1.740617	-3.00137	0.002688	0.008254
SS1G_13444	4.23214	-5.63698	1.929605	-2.92131	0.003486	0.010371
SS1G_00683	7.260228	-4.95318	1.765727	-2.80518	0.005029	0.014248
SS1G_05338	4.674965	-5.78335	2.284276	-2.53181	0.011348	0.02892

Table S14. RT-qPCR primer design

Description	GeneID	Forward	Reverse
Actin-REF	SS1G_08733	AGATCTTGGCTGAGCGTGGTTACA	TGACTGGCGGTTTGGATTTCTTGC
ago-4	SS1G_11723	TTATGGATGAAGCCCAGGTC	TGGTAGAGCGCATGTACTGG
Cellobiohydrolase	SS1G_04945	GATGTCTGGGAAGCCAACTC	CACCAGTGCAAGCAGTTTGT
DNA Pol ε subunit 2	SS1G_05217	TTGACTTTGACGTCGTCTGC	CCTTCTTCCCTCCATCCTGT
Mcm4	SS1G_01278	CTGCAAAGCTGGTGACAGAG	GAGTCCGTTGACGTGGATTT
Putative DSS1	SS1G_01104	CGTTGAGAACTGGAGCCAAG	CATCGTCATCCCAGCTCTCT
Putative PCNA	SS1G_12686	AATGGTGCTGTTACCCTTCG	AAGGAGACCGGTTCGGTAAG
QDE1	SS1G_03377	CCCTAGCGGACAAATTATCG	ACTCTCAACGATGGCAGGAT
RNase H2 subunit C	SS1G_13856	GTCCACCATCCAAACAATCC	TGGGTGCAGTGTGGTTTATG
RRP3	SS1G_09915	GAAGCTTTCAACGCAAGAGG	CTCGATGAACCAGAGCAGTG
SAD1	SS1G_13161	ATTGGCTGTGGAGGAATCTG	TTAGAGCCGGTGGAGAGAAA
TOP3	SS1G_12655	TTCACACGCTTTCAGACGAC	TGACACGACCCATAGCTCAG
Ubiquitin-conjugating enzyme E2 H	SS1G_02279	AACTTGGTCGCCTATGTTCG	CCGTGGGATTTGGATATCTG
β-fructofuranosidase	SS1G_07184	CTCTAGCTGGTGCCCTTCTG	CCTGGGATAGGTGCATCAGT

Table S15. RT-qPCR results

Target	Bio Group	Control	Expression	Expression 95% Cl Low	Expression 95% Cl High	*p*-Value
Actin-REF	VF	C	N/A	N/A	N/A	N/A
Actin-REF	VI		N/A	N/A	N/A	N/A
ago4	VF	C	1.00000	0.35009	2.85643	N/A
ago4	VI		0.43167	0.03125	5.96259	0.380895
B-fructofrano	VF	C	1.00000	0.50825	1.96755	N/A
B-fructofrano	VI		1.40563	0.27385	7.21484	0.562905
Cellobiohydro	VF	C	1.00000	0.26797	3.73179	N/A
Cellobiohydro	VI		1.74191	0.14422	21.03867	0.553905
DSS1	VF	C	1.00000	0.25982	3.84886	N/A
DSS1	VI		0.99284	0.31992	3.08118	0.990052
MCM4	VF	C	1.00000	0.31369	3.18787	N/A
MCM4	VI		1.02862	0.01964	53.87070	0.983337
PCNA	VF	C	1.00000	0.31233	3.20176	N/A
PCNA	VI		1.15122	0.07931	16.71109	0.882949
Pol-e	VF	C	1.00000	0.29726	3.36405	N/A
Pol-e	VI		0.43810	0.00086	222.11502	0.551193

Target	Bio Group	Control	Expression	Expression 95% Cl Low	Expression 95% Cl High	*p*-Value
QDE1	VF	C	1.00000	0.21638	4.62151	N/A
QDE1	VI		0.45557	0.04407	4.70888	0.404776
RNase H2C	VF	C	1.00000	0.39013	2.56325	N/A
RNase H2C	VI		0.78842	0.08261	7.52474	0.767408
RRP3	VF	C	1.00000	0.35709	2.80042	N/A
RRP3	VI		0.80875	0.06067	10.78112	0.816577
SAD1	VF	C	1.00000	0.50863	1.96607	N/A
SAD1	VI		0.35947	0.00446	28.96856	0.491102
TOP3	VF	C	1.00000	0.18504	5.40418	N/A
TOP3	VI		1.46181	0.15082	14.16841	0.684242
Ubiquitin E2H	VF	C	1.00000	0.19435	5.14545	N/A
Ubiquitin E2H	VI		1.30023	0.61619	2.74361	0.658937
Ubiquitin-REF	VF	C	1.00000	0.45682	2.18906	N/A
Ubiquitin-REF	VI		1.00598	0.35062	2.88629	0.988939
